# Single-molecule mitochondrial DNA imaging reveals heteroplasmy dynamics shaped by developmental bottlenecks and selection *in vivo*

**DOI:** 10.1016/j.devcel.2026.03.011

**Published:** 2026-05-13

**Authors:** Rajini Chandrasegaram, Sara Gottardo, Abhilesh Dhawanjewar, Antony M. Hynes-Allen, Beitong Gao, Suvagata Roy Chowdhury, Luis-Carlos Tábara, Michele Frison, Stavroula Petridi, Patrick F. Chinnery, Julien Prudent, Hansong Ma, Jelle van den Ameele

**Affiliations:** 1 MRC Mitochondrial Biology Unit, University of Cambridge, Cambridge CB2 0XY, UK; 2 Department of Clinical Neurosciences, University of Cambridge, Cambridge CB2 0XY, UK; 3 Department of Genetics, University of Cambridge, Cambridge CB2 3EH, UK; 4 School of Biosciences, University of Birmingham, Birmingham B15 2TT, UK

## Abstract

Mitochondrial DNA (mtDNA) exists in many copies per cell, with cell-to-cell variability in mutation load, which is known as heteroplasmy. Developmental and age-related expansion of heteroplasmic mtDNA mutations contributes to the pathogenesis of mitochondrial and neurodegenerative diseases. Here, we describe an approach for *in situ* sequence-specific detection of single mtDNA molecules (mtDNA-single-molecule fluorescent *in situ* hybridization [smFISH]). We apply this method to visualize and measure mtDNA and heteroplasmy levels *in situ* at single-cell resolution in whole-mount *Drosophila* tissue and cultured human cells. In *Drosophila*, we identify a somatic mtDNA bottleneck during neurogenesis. This amplifies heteroplasmy variability between neurons, as predicted by a mathematical bottleneck model, predisposing individual neurons to a high mutation load. However, both during neurogenesis and oogenesis, mtDNA segregation is accompanied by purifying selection, promoting wild-type (WT) over pathogenic mtDNA. mtDNA-smFISH thus elucidates how developmental cell-fate transitions, accompanied by changes in cell morphology, behavior, and metabolism, can shape the transmission and selection of deleterious mtDNA variants.

## Graphical abstract

**Graphical abstract:**
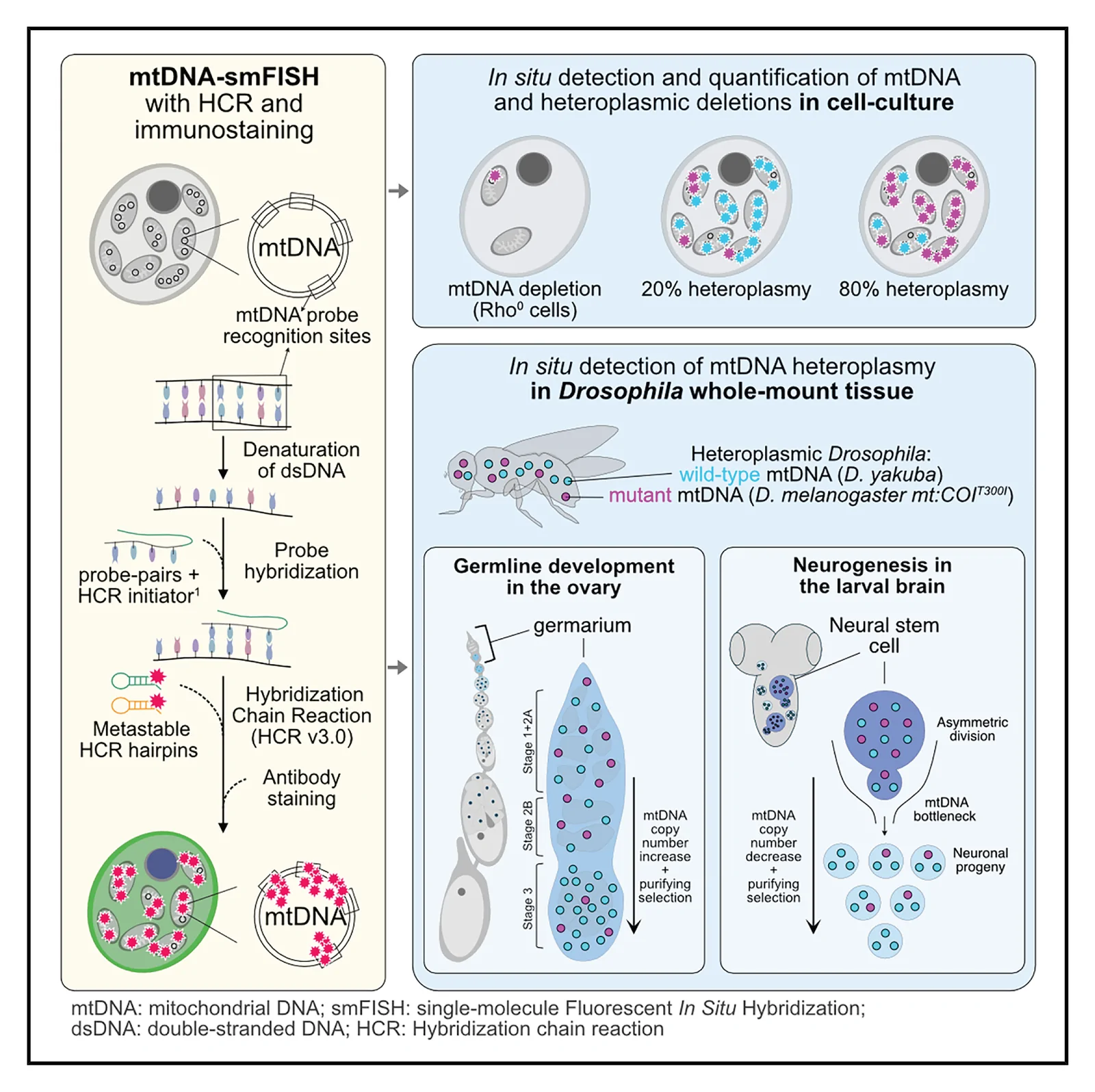

## Introduction

Unlike the nuclear genome, which is haploid or diploid in most animal cells, the mitochondrial genome (mtDNA) occurs in up to many thousands of copies per cell. Most human cells carry a mixture of wild-type (WT) and mutated mtDNA (heteroplasmy),^1–6^ with considerable cell-to-cell variability in mutation load.^7,8^ During development and aging, mtDNA mutations can clonally expand to high levels in single cells, causing defects in energy production and metabolism and eventually cell death.^5,9–11^ Expansion of pathogenic mtDNA mutations follows striking tissue- and cell-type-specific patterns during development and aging,^10,12^ and is the main cause of inherited and sporadic adult-onset mitochondrial diseases.^13^ Clonal amplification of mtDNA mutations has also been implicated in common neuro-degenerative disorders, such as Alzheimer’s and Parkinson’s disease,^14,15^ and in cancer, in which certain somatic mtDNA variants reach homoplasmy due to positive selection.^16^ In *Drosophila*, deleterious mtDNA mutations have also been shown to increase within individual flies over time,^17–19^ contributing to developmental and aging phenotypes and even lethality.^19–25^

Variability in heteroplasmy levels between cells within a tissue or between a mother and her offspring can be caused by vegetative segregation. This process is driven by stochastic mechanisms, primarily the random partitioning of mtDNA during cell division and the turnover of mtDNA molecules due to ongoing destruction and replication of mtDNA independent of the cell cycle (relaxed replication).^7,9^ These processes lead to heteroplasmy drift and may be modulated by purifying selection or selfish propagation, resulting, respectively, in a decrease or an increase in deleterious mtDNA heteroplasmy levels.^6,11,18^ A special example occurs between mothers and their offspring, in which heteroplasmy shifts are further accentuated by a genetic mtDNA bottleneck caused by mtDNA copy number (CN) reduction in single cells during maternal germline development.^26–31^ Although well studied in the germline, little is known about the segregation of heteroplasmy in somatic tissues during development and aging, apart from observations from mtDNA analysis in hair follicles.^32–34^ Nonetheless, this is likely to play a crucial role in determining mutational burden across tissues throughout life. For example, many developmental and homeostatic cell-fate transitions are accompanied by profound changes in cell volume, morphology, and metabolism, but it remains unknown how these affect mtDNA and whether they may amplify shifts in heteroplasmy levels.

Current explanations of heteroplasmy shifts are mainly based on modeling of data from whole-tissue analysis^35^ or single-cell sequencing and genotyping of isolated cells outside their *in vivo* context.^7,8,32,36–38^ A major limitation of these approaches is that they mostly rely on tissue dissociation, thereby losing spatial information and limiting the analysis of subcellular mechanisms.

Here, we develop and validate an approach for the *in situ* visualization of heteroplasmic mtDNA variants (mtDNA-single-molecule fluorescent *in situ* hybridization [smFISH]) based on smFISH combined with enzyme-free hybridization chain reaction (HCR) for signal amplification. We apply this method to detect mtDNA heteroplasmy *in situ* in whole-mount tissue from an established *Drosophila* model of stable heteroplasmy and in cultured human cybrid cells carrying a heteroplasmic single large-scale mtDNA deletion. In *Drosophila*, mtDNA-smFISH confirmed the presence of purifying selection against pathogenic mtDNA during oocyte development. In addition, we provide evidence for the existence of a somatic bottleneck during neurogenesis in the developing larval brain. This amplifies cell-to-cell variability in heteroplasmy levels between different progeny derived from the same neural stem cell (NSC), thereby predisposing subsets of neurons and glia to high levels of deleterious mtDNA mutations, which can lead to mitochondrial dysfunction and disease.

### Design

Current mtDNA imaging methods either lack the resolution to distinguish single mtDNA molecules,^39,40^ have low sensitivity,^17,41^ depend on mtDNA replication,^42^ rely on cell penetration of enzymes and have not been used on whole-mount tissues,^43,44^ or are not compatible with the genetics of multicellular organisms.^45^ Development of *in vivo* models and tools to measure, track, and modulate heteroplasmy in somatic tissues is required to understand the cellular and molecular processes that cause somatic heteroplasmy variation and tissue-specific phenotypes of mitochondrial disease and to answer long-standing questions in the field of mitochondrial/mtDNA biology.^46^

**Figure 1:**
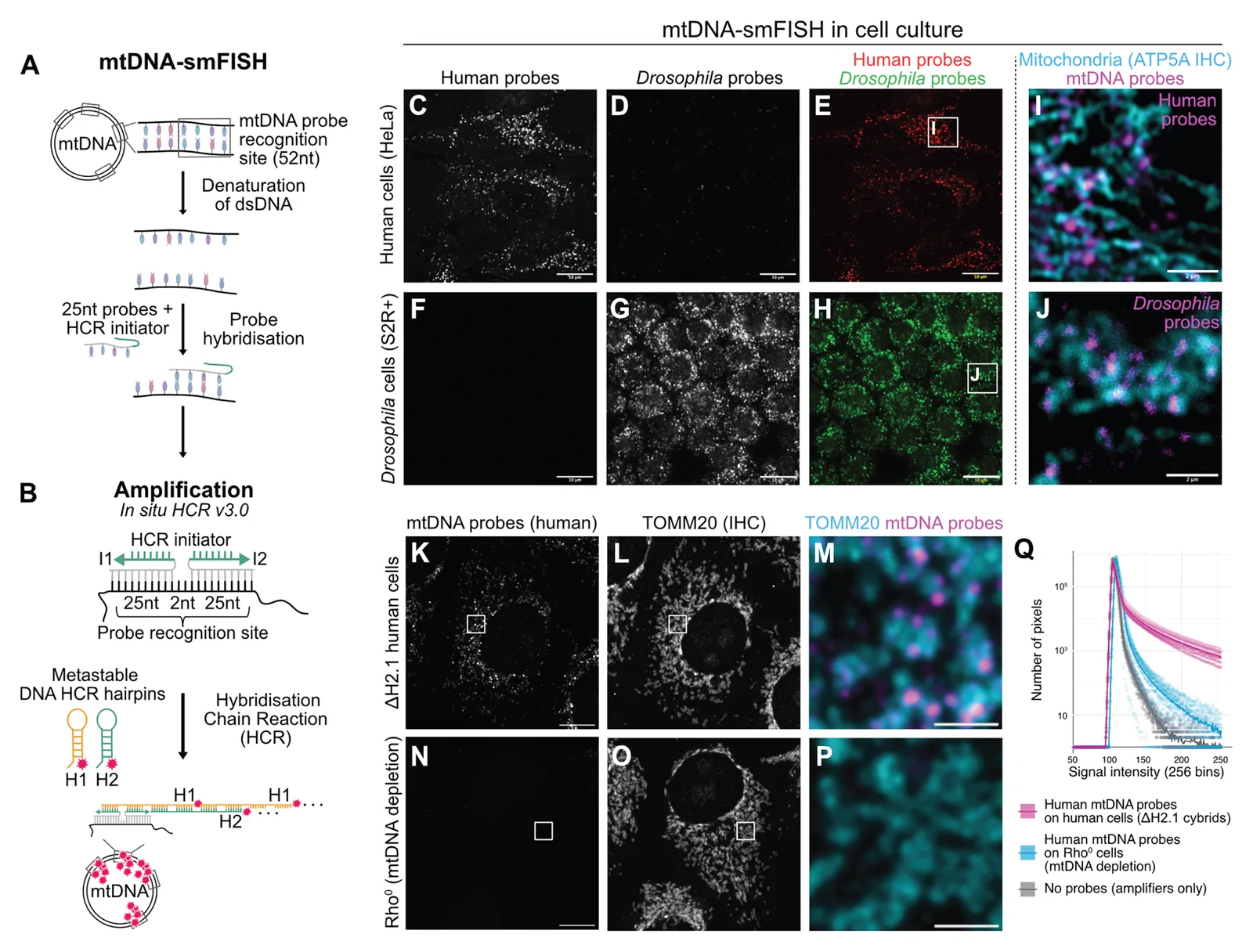
Single-molecule fluorescent *in situ* hybridization of mtDNA in cell culture (A) Pairs of 25-nucleotide (nt) probes were designed against 52-nt mtDNA target sequences and used for *in situ* hybridization after denaturation. (B) Signal amplification relied on metastable DNA hairpins recognizing an initiator sequence formed when a probe pair hybridizes on the target sequence, initiating an enzyme-free hybridization chain reaction (HCR). (C–H) Representative images of mtDNA-smFISH in human HeLa cells (Airyscan imaging, C–E) or *Drosophila* S2R+ cells (confocal imaging, F–H) with probes against human (C, E, F, and H) or *Drosophila* (D, E, G, and H) mtDNA. (I and J) Zoomed areas boxed in (E) and (H) showing mtDNA-smFISH (magenta) followed by co-immunostaining (cyan) against the mitochondrial marker ATP5A1. (K–Q) mtDNA-smFISH (K–P) and signal quantification (Q, experiment 3 in Figures S2H–S2J) with probes against human mtDNA and co-immunostaining with the mitochondrial marker TOMM20 in a ΔH2.1 cybrid cell-line (low percentages of deleted mtDNA, K–M) and the same cell-line with mtDNA depletion (Rho^0^) upon ddC treatment (N–P). Zoomed areas (M and P) boxed in (L) and (O) show mtDNA-smFISH (magenta) followed by co-immunostaining (cyan) against TOMM20. Scale bars are 10 μm (C–H, K and N) and 2 μm (I, J, M, and P). See also Figures S1 and S2 and Table S1.

To develop an approach for *in situ* detection of heteroplasmic mtDNA variation in whole-mount tissues, we took advantage of recent advances in smFISH combined with HCR. smFISH-HCR is a well-established technique for the detection of mRNA in whole-mount tissues.^47–50^ It combines short single-stranded DNA probes complementary to target nucleotide sequences with enzyme-free signal amplification based on fluorescently labeled metastable DNA hairpins (Figure 1A). Third-generation smFISH-HCR achieves high signal-to-background ratios by relying on pairs of short (25 bp) single-stranded DNA probes that together recognize a 52 bp mRNA target sequence and initiate HCR only when bound in close proximity and correct orientation^47^ (Figure 1B). This allows *in situ* single-molecule detection with as few as 5 probe pairs or less.^50^ This approach has been highly successful and widely adopted for RNA-smFISH because of its versatility and ease, particularly for whole-mount samples. To detect mtDNA instead of mRNA, we established a protocol for double-stranded DNA (dsDNA) denaturation in whole-mount tissue through heating of formaldehyde-and methanol-fixed tissue to 95°C in 70% formamide (see STAR Methods). This allows double-stranded mtDNA to become single stranded, enabling the probes to anneal to their complementary mtDNA sequence *in situ* and subsequently recruit fluorescent hairpins, which are visualised by fluorescence microscopy.

## Results

### Single-molecule FISH of mtDNA

We first designed sets of 12 and 10 probe pairs to specifically detect human or *Drosophila* mtDNA, respectively (Figure S1; Table S1). We used these to conduct mtDNA-smFISH on human or *D. melanogaster* cells cultured *in vitro*. Each probe set generated a distinct cytoplasmic punctate staining pattern in the cells of its corresponding species (human or *Drosophila*) (Figures 1C–1J). In contrast, when the same probe sets were applied to the converse cell types (i.e., human probes on *Drosophila* cells and vice versa), no signal was detected (Figures 1D and 1F).

We next quantified the signal intensity of the human probe set in Rho^0^ cells (derived from ΔH2.1 cell lines carrying a 7.5 kb mtDNA deletion; see STAR Methods) after ddC-mediated depletion of mtDNA (Figures S2A–S2M). The mtDNA-smFISH signal intensity showed cell-to-cell variability in the Rho^0^ cell cultures upon ddC treatment (Figures S2I and S2K–S2M), corroborating the capacity of the method to monitor single-cell mtDNA levels. Signal intensity compared with non-treated cells decreased progressively with continuous ddC treatment (Figure S2I), reaching levels that were only slightly higher than the background signal obtained from mtDNA-smFISH conducted with amplifiers only (i.e., without any probes) (Figures 1Q, S2I, and S2J). In contrast, RNase treatment before hybridization did not prevent the mtDNA-smFISH staining (Figures S2N–S2Q), altogether indicating the specificity of the probes for mtDNA.

To further confirm that the punctate pattern corresponded to mtDNA, we conducted fluorescent immunohistochemistry (IHC) with antibodies against mitochondrial epitopes that with-stand the harsh denaturation conditions of mtDNA-smFISH. Co-IHC of ATP synthase (ATP5A1; Figures 1I and 1J), localized at the inner mitochondrial membrane, or of the outer mitochon-drial membrane protein TOMM20 (Figures 1K–1P), together with mtDNA-smFISH, showed a clear overlay of smFISH puncta with the mitochondrial network, both in human and *Drosophila* cells. A gap in the ATP synthase staining was sometimes observed at the site of mtDNA-smFISH by Airyscan super-resolution microscopy (Figures S2R–S2V). This may be due to steric hindrance from the HCR hairpins, preventing efficient antibody access.^51,52^ However, a similar lack of overlap between ATP synthase IHC and mtDNA nucleoids has been described previously using other approaches,^53–55^ suggesting that mtDNA nucleoids may be situated away from mitochondrial cristae or the ATP synthase complex within those cristae. Finally, we combined mtDNA-smFISH with IHC of the nucleoid-associated mito-chondrial transcription factor A (TFAM).^56–58^ TFAM IHC was more diffuse than the mtDNA-smFISH signal and showed colocalization with 78.9% ± 9.4% (mean ± SD) of mitochondrial-localized mtDNA-smFISH puncta (Figures S2W–S2Y). The lack of complete overlap is likely due to variability in TFAM binding and nucleoid organization, in line with recent results from long-read single-molecule accessibility mapping.^57,58^

### mtDNA-smFISH in whole-mount tissue

**Figure 2:**
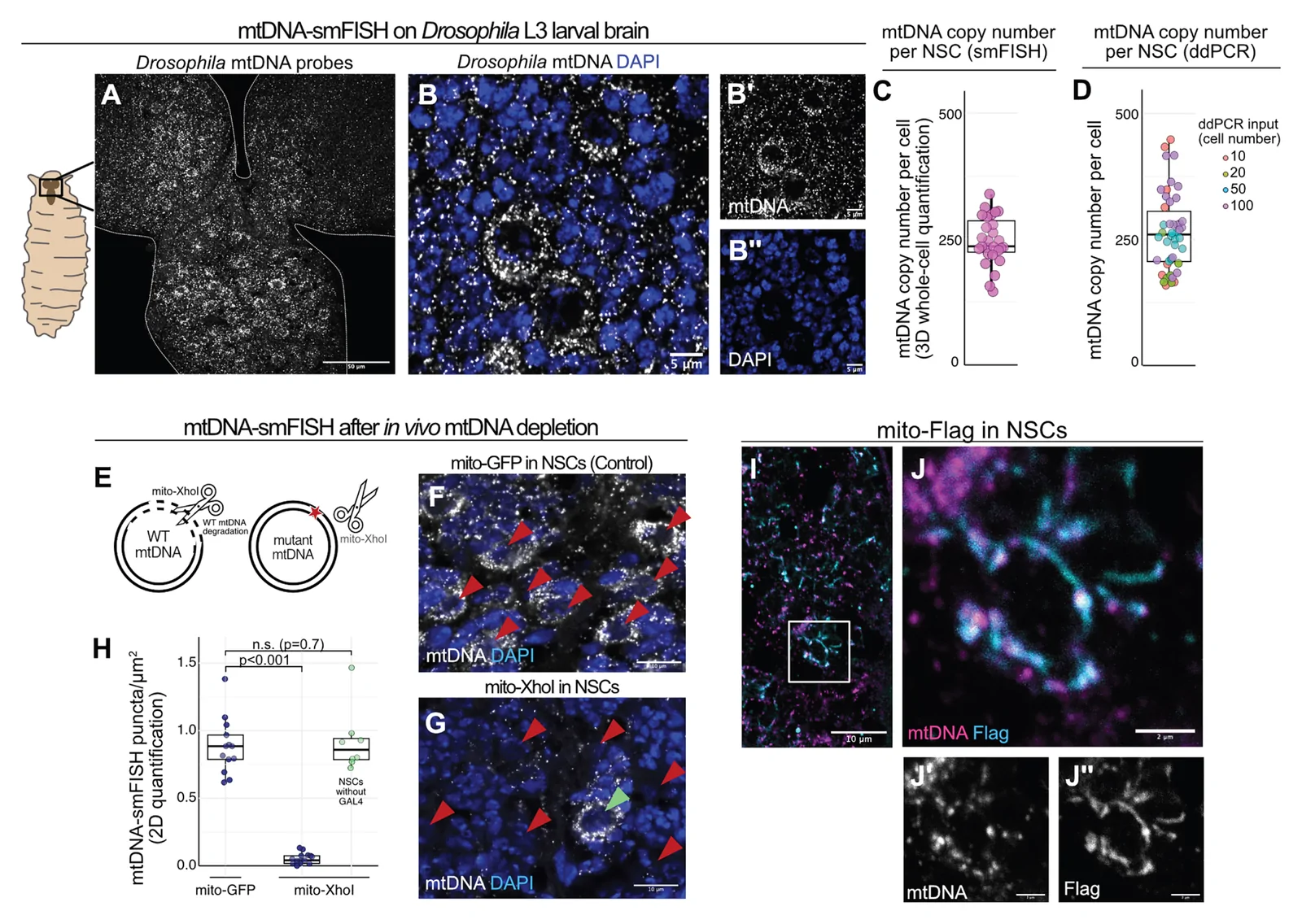
mtDNA-smFISH in whole-mount *Drosophila* tissue (A and B–B″) Representative confocal images of mtDNA-smFISH (gray) in the *Drosophila* third instar larva brain and co-labeling with DAPI (blue) to mark nuclei. Single channels of (B) are shown in (B′) and (B″). (C and D) mtDNA CN quantification in complete single NSCs by mtDNA-smFISH across the 3D z stack (C) or by ddPCR (D), with each dot representing mtDNA CN in a single NSC. (E) Schematic of mito-XhoI activity resulting in digestion and degradation of wild-type (WT) but not mutant mtDNA. (F–H) Mito-GFP control (F) or mito-XhoI (G) expression in NSCs (*worniu*-GAL4) of homoplasmic WT *D. melanogaster* larval brains, followed by mtDNA-smFISH (gray) and DAPI counterstaining (blue). Red arrowheads indicate NSCs, and the green arrowhead indicates an NSC that does not express *worniu*-GAL4 and therefore retains WT mtDNA. Quantification of mtDNA-smFISH puncta in 2D confocal sections of NSCs, presented as puncta per μm^2^ (H) (Mann-Whitney U test with Holm-Bonferroni multiple-test correction; *n* = 13 NSCs from 5 brains [mito-GFP] and 26 NSCs from 3 brains [mito-XhoI]). (I and J–J″) mtDNA-smFISH (magenta) and immunostaining (cyan) against FLAG in NSCs expressing a mitochondrial-targeted FLAG-tagged transgene. Single channels of (K) are shown in (K′) and (K″). Scale bars are 50 μm (A), 10 μm (F, G, and J), 5 μm (B), and 2 μm (K). See also Figures S3 and S4.

We next applied mtDNA-smFISH to whole-mount *Drosophila* larval brains and observed a similar cytoplasmic punctate staining pattern throughout the entire brain (Figures 2A and 2B). No signal was observed under conditions without probes or amplifiers (Figures S3A–S3F), and the probes recognized mtDNA from both *D. yakuba* (Dyak) or *D. melanogaster* (Dmel) strains, as they were designed against conserved regions of the two mitochondrial genotypes (Figures S1 and S3G–S3I). To estimate sensitivity, we quantified smFISH puncta in single NSCs, which can be identified within the larval ventral nerve cord (VNC) by their large nuclear size and expression of green fluorescent protein (GFP) under the control of an NSC-specific GAL4.^59,60^ Quantification was performed in 3D in z stacks across individual NSCs and compared with mtDNA CN quantification by single-cell digital droplet PCR (ddPCR) (Figures S4A–S4H) of fluorescence-activated cell sorting (FACS)-sorted nuclear GFP-labeled NSCs (Figures S4I–S4N). The average NSC mtDNA CN detected by smFISH was 246.1 ± 47.4 (mean ± SD) (Figure 2C), and 259.6 ± 76.1 (mean ± SD) by ddPCR (Figure 2D), indicating >90% efficiency of mtDNA-smFISH in detecting mitochondrial nucleoids across experiments. The difference between the two techniques may reflect mtDNA replication occurring in some nucleoids, which are therefore expected to contain >1 copy of mtDNA per mtDNA-smFISH punctum.^58^ Importantly, these quantifications indicate that, also *in vivo* and in non-mammalian species, the majority of nucleoids harbor only a single copy of mtDNA, in line with previous observations from *in vitro* studies with cultured mammalian cells.^58,61^

Expression of a mitochondrial-targeted restriction enzyme, mito-XhoI, in *Drosophila* cells was previously shown to linearize mtDNA through digestion at a single restriction site (Figure 2E), resulting in mtDNA degradation and depletion.^62^ When we expressed mito-XhoI specifically in NSCs and their progeny with *worniu*-GAL4, the mtDNA-smFISH signal was significantly reduced (Figures 2F–2H), similar to what was observed for the human probe set in mtDNA-depleted Rho^0^ cells (Figures 1K–1Q). Only glial cells and NSC lineages not expressing *worniu-* GAL4 retained mtDNA-smFISH signal (Figures 2F and 2H).

Co-IHC of a mitochondrial-targeted FLAG-tagged transgene expressed in NSCs showed overlap with mtDNA-smFISH signals (Figures 2I and 2J), further confirming the specificity of mtDNA-smFISH. Moreover, mtDNA-smFISH performed reliably in other developing and adult *Drosophila* tissues, including the wing imaginal discs, larval salivary gland, thoracic muscle, and ovaries (Figures S3J–S3M). Together, these results demonstrate that mtDNA-smFISH can specifically label mtDNA molecules *in situ* with single-molecule resolution not only in human and *Drosophila* cell culture but also in whole-mount tissues, thus opening the possibility for future studies of genetic mtDNA variation at subcellular or single-mitochondrion resolution.

### Immunofluorescence combined with mtDNA-smFISH

**Figure 3:**
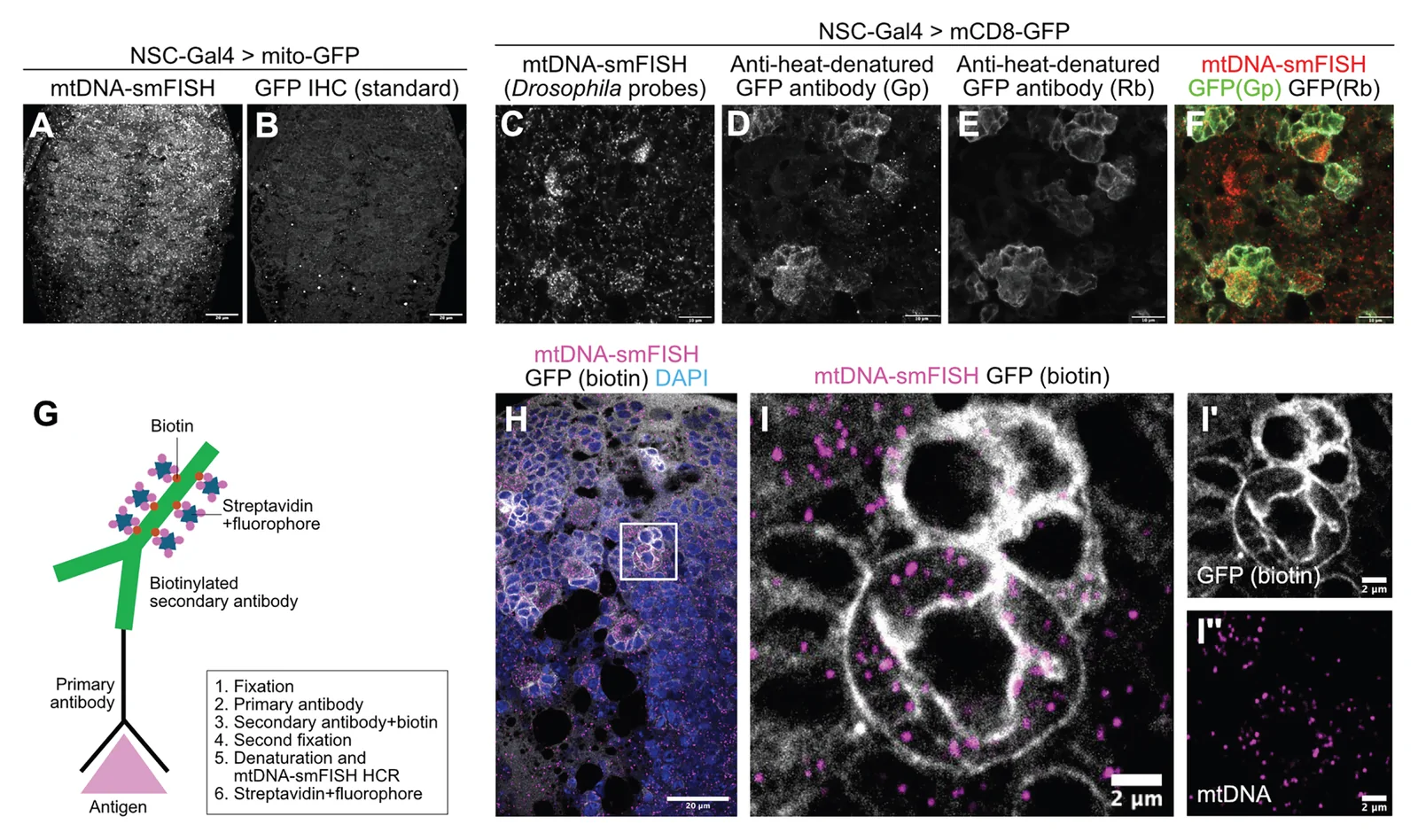
Immunofluorescence combined with mtDNA-smFISH (A and B) mtDNA-smFISH (A) and conventional immunostaining for GFP (*worniu*-GAL4 > UAS-mito-GFP) (B) showing no effective GFP staining due to the high denaturation temperature required for mtDNA-smFISH. (C–F) Representative confocal images of mtDNA-smFISH (C) with immunostaining using anti-heat-denatured GFP antibodies raised in guinea pig (D) or rabbit (E) to mark NSCs (*worniu*-GAL4 > UAS-mCD8-GFP) in the *Drosophila* larval brain. (G) Schematic of biotin-labeled antibody immunostaining to circumvent denaturation of antigens. (H and I–I″) mtDNA-smFISH (magenta) combined with biotin-labeled antibody staining of mCD8-GFP in NSCs (*worniu*-GAL4, gray) and DAPI counterstain (blue). Single channels of (I) are in (I′) and (I″). Scale bars are 50 μm (A and B), 20 μm (H), 10 μm (C–F), and 2 μm (I).

Although we could successfully conduct anti-ATP5A1 (Figures 1I–1N), anti-TOMM20 (Figures 1K–1P), anti-TFAM (Figures S2W–S2Y), and anti-FLAG-tag (Figures 2I and 2J) IHC after the harsh denaturation conditions of mtDNA-smFISH, this was not the case for most other epitopes, such as GFP (Figures 3A and 3B). Many epitopes do not tolerate denaturation because they change conformation upon fixation, heating, or formamide treatment. Although specific anti-heat-denatured antibodies exist for GFP,^63^ allowing anti-GFP immunostaining in whole-mount tissues after denaturation (Figures 3C–3F), such reagents are not available for most proteins or epitopes. We therefore developed a protocol that includes immunostaining with biotin-labeled antibodies prior to denaturation (Figure 3G), as biotin remains intact during the denaturation step. When combined with a second round of fixation after primary antibody incubation (see STAR Methods), this allows visualization of the primary antibody binding pattern with a streptavidin-conjugated fluorophore post-mtDNA-smFISH (Figures 3H and 3I).

### mtDNA-smFISH to detect heteroplasmic mtDNA deletions

**Figure 4:**
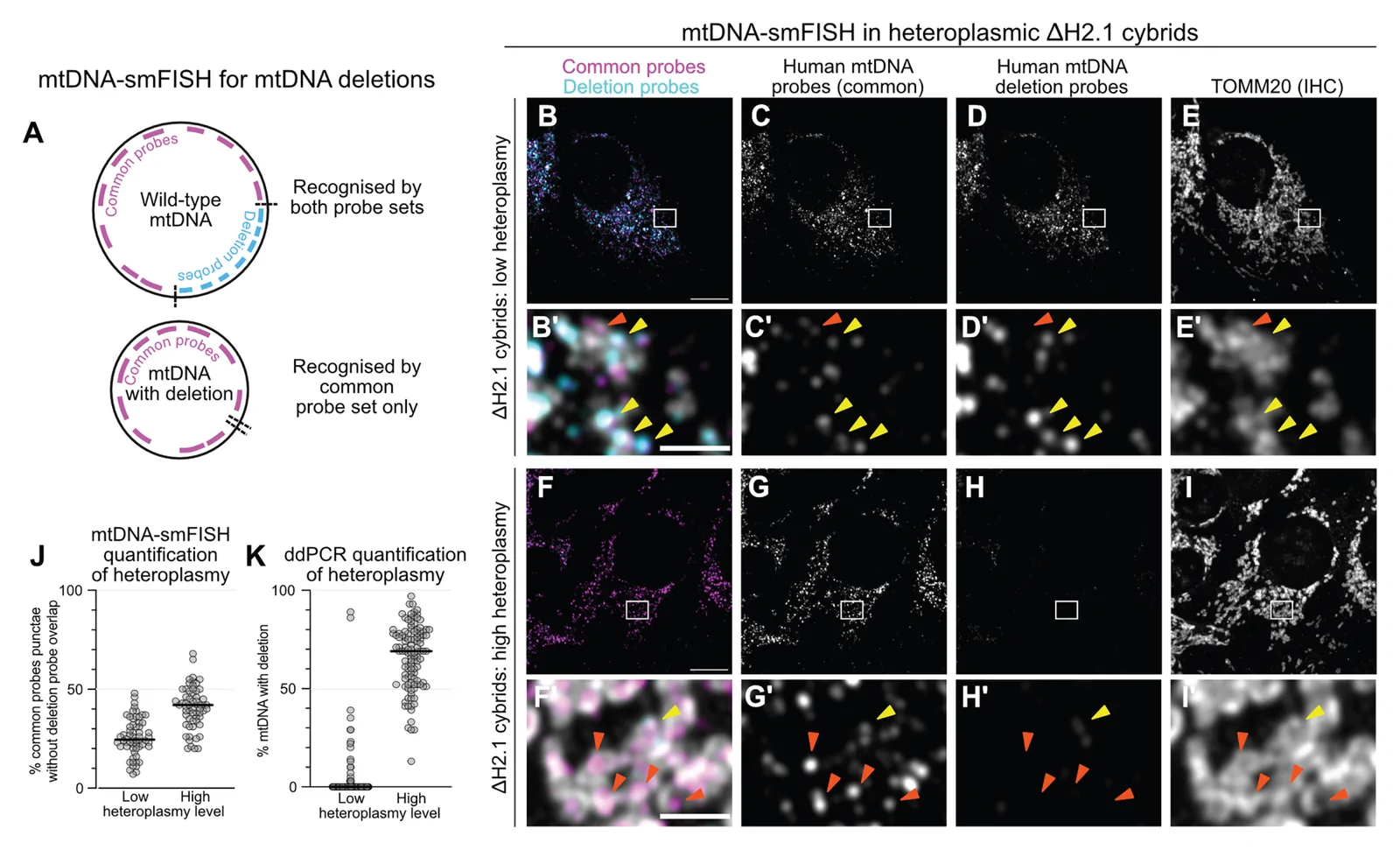
Visualizing mtDNA deletion heteroplasmy with mtDNA-smFISH in cell culture (A) Schematic of mtDNA-smFISH for detection of single mtDNA deletions. (B–I′) Representative confocal images of mtDNA-smFISH in ΔH2.1 cybrid cells with probes against all human mtDNA molecules (magenta; C and G) or only against the deleted region (cyan; D and H), with TOMM20 counterstain (E and I). Note that mtDNA deletion probes recognize only molecules that do not carry the deletion, and a reduced signal indicates higher levels of mutant mtDNA carrying the deletion in a cell. Zoomed regions boxed in (B)–(I) are shown in (B′)–(I′); red arrowheads point to deleted mtDNA and yellow arrowheads to WT mtDNA. (J and K) Heteroplasmy levels (% deleted mtDNA) in single ΔH2.1 cybrid cells from low- or high-heteroplasmy clonal cultures, quantified in 2D on maximum-intensity projection after mtDNA-smFISH (J) or by ddPCR (K). 60 cells were analyzed across three independent experiments; the line indicates the mean. Scale bars are 5 μm (B and F) and 2 μm (B′ and F′). See also Figure S5 and Table S1.

Having established mtDNA-smFISH for sequence-specific *in situ* detection of single mtDNA molecules, we next asked whether this approach would allow us to distinguish different mtDNA molecules within the same cell. mtDNA deletions have previously been detected *in situ* in cultured human cells by FISH using long probes.^40^ We adapted mtDNA-smFISH for the same purpose, to detect mtDNA heteroplasmy in human cybrid cells carrying a mixture of WT mtDNA and mtDNA molecules with a single large deletion (ΔH2.1).^64^ We designed one set of 12 probe pairs to specifically label full-length, WT mtDNA molecules by targeting the mtDNA deletion sequence (“deletion probes”), in addition to the human probe set we initially designed, recognizing all (i.e., deleted and full-length) mtDNA molecules (“common probes”) (Figures 4A and S1; Table S1). The specificity of all individual probe pairs was tested by conducting mtDNA-smFISH with each probe pair at 100× concentration in cybrid cells before and after ddC-mediated mtDNA depletion (Rho^0^ cells) (Figure S5). This showed substantial differences in signal intensity between probe pairs (Figures S5A–S5C, S5G, and S5H). The intensity obtained in mtDNA-depleted Rho^0^ cells was consistently lower than that in non-depleted cells (Figures S5D–S5H), except for one probe pair (common probe 3; Figure S5G; Table S1), which was removed for further experiments.

We conducted mtDNA-smFISH using both human probe sets recognizing either the deleted or non-deleted sections of human mtDNA for mtDNA-smFISH in ΔH2.1 cells with either low (Figures 4B–4E) or high (Figures 4F–4I) heteroplasmy. Single-cell heteroplasmy levels were quantified as the percentage of mitochondrial-localized (TOMM20-positive) puncta detected by the common probe set that did not overlap with signals from the deletion probe set (Figure S1H), using a custom-made FiJi plugin for automated heteroplasmy quantification in 2D (see STAR Methods). Because mtDNA-smFISH quantification was performed in 2D and detection of deleted molecules relies on a negative measurement (i.e., the absence of signal from the deletion probes) (Figure 4A), we cannot yet directly relate absolute single-cell heteroplasmy quantification obtained by mtDNA-smFISH to ddPCR-based measurements without prior quantification of sensitivity for both probe sets. Nevertheless, mtDNA-smFISH could distinguish low- from high-heteroplasmy cell cultures (Figure 4J; 25.8% ± 9.5% [mean ± SD] for low and 40.8% ± 10.6% [mean ± SD] for high heteroplasmy), as confirmed by ddPCR-based quantification of single-cell heteroplasmy levels from the same cultures (Figure 4K; 3.8% ± 13.2% [mean ± SD] for low and 66.2% ± 16.1% [mean ± SD] for high heteroplasmy).

### mtDNA-smFISH to detect mtDNA heteroplasmy *in vivo*

**Figure 5:**
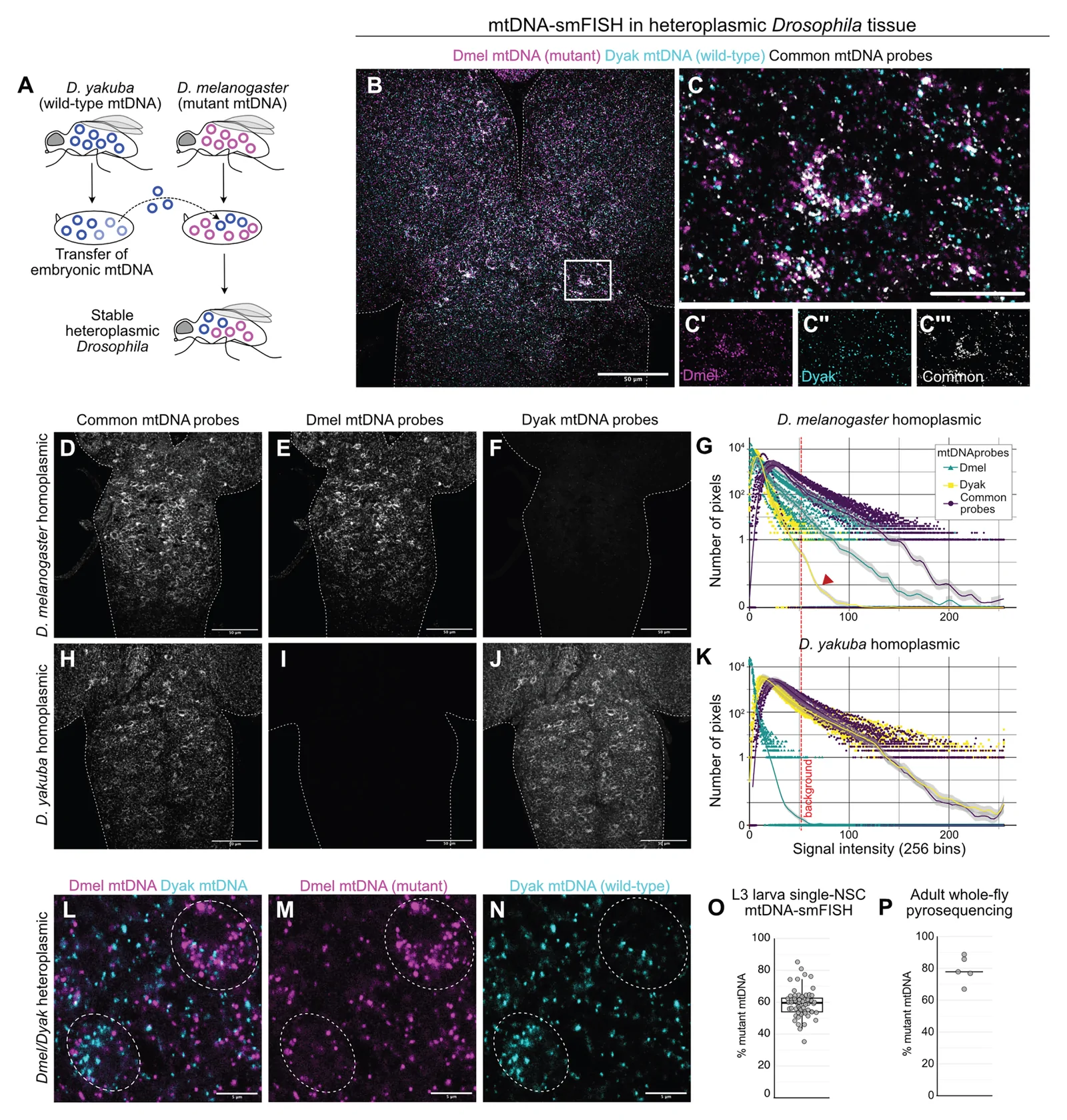
Visualizing heteroplasmy with mtDNA-smFISH whole-mount *Drosophila* tissue (A) Generating heteroplasmic *Drosophila* through mtDNA transfer between different species. (B and C) mtDNA-smFISH using probes targeting only *D. melanogaster* (mutant, magenta), only *D. yakuba* (WT, cyan), or mtDNA from both species (common, gray) in heteroplasmic *Drosophila* brains. (D–K) Representative confocal images of mtDNA-smFISH (D–F and H–J) and signal quantification (G and K; mean ± SEM) with probes specific to either *D. melanogaster* (E and I, green graphs) or *D. yakuba* (F and J, yellow graphs) mtDNA, or common to both (D and H, purple graphs), in L3 larval brains homoplasmic for *D. melanogaster* (D–G) or *D. yakuba* (H–K) mtDNA. The red dashed line in the graphs separates the true signal from the background, with a threshold set at 55 bins (see also Figures S6A and S6B). (L–P) Heteroplasmy levels (% mutant mtDNA) in single NSCs of heteroplasmic L3 larval brains quantified after mtDNA-smFISH (O), with representative confocal images in (L)–(N), or in whole adult flies determined by pyrosequencing (P). Scale bars are 50 μm (B and D–J), 10 μm (C), and 5 μm (L–N). See also Figure S6 and Table S1.

To study mtDNA heteroplasmy *in vivo*, we took advantage of a heteroplasmic Dmel fly line that stably transmits a temperature-sensitive lethal mtDNA mutation in the COI subunit of the cytochrome oxidase complex (*mt:COI*^*T300I*^) together with a functionally neutral 9 bp deletion (*mt:ND2*^*del1*^) in the ND2 subunit of Complex I.^62^ The *mt:COI*^*T300I*^ mutation impairs cytochrome oxi-dase activity at higher temperature (29°C), causing lethality at late pupal stages in homoplasmic flies.^24,65^ Introducing WT mtDNA from Dyak species through germ-plasm transfer^66^ rescues viability in *mt:COI*^*T300I*^ flies.^18,39,67^ As a result, these *D. melanogaster* flies (i.e., with a *D. melanogaster* nuclear genome) carry both the Dmel *mt:COI*^*T300I*^ mutant and Dyak WT mtDNA at high levels of heteroplasmy (Figures 5A, S1I, and S1J).

We designed mtDNA-smFISH probes to distinguish Dmel (carrying the *mt:COI*^*T300I*^ mutation) from Dyak (WT) mtDNA (Figure S1; Table S1). The main sequence divergence between Dmel and Dyak mtDNA lies in the non-coding D-loop, allowing FISH with long probes,^39^ but precluding single-molecule analysis. Due to its repetitive and AT-rich nature, this non-coding region is not amenable to smFISH with small probes. We therefore designed 7 (Dmel) and 5 (Dyak) probe pairs across the most divergent coding regions characterized by small indels or multiple single-nucleotide polymorphisms (Figure S1C). mtDNA-smFISH in heteroplasmic larval brains yielded clear punctate cytoplasmic staining for both probe sets (Figures 5B and 5C), albeit weaker than mtDNA-smFISH with the common probe set consisting of 10 probe pairs (Figures 5D–5K).

As with the other probe sets, no signal was detected in conditions without probes or amplifiers (Figures S6A and S6B). Testing specificity on brains from WT homoplasmic Dmel or Dyak flies (i.e., only containing WT *D. melanogaster* or WT *D. yakuba* mtDNA^68^) showed that the Dmel probe set produced no signal in Dyak tissue (Figures 5D–5K). Some punctate staining was observed with the Dyak probe set on homoplasmic Dmel tissues (Figures 5F and 5G), which was not due to any specific probe pair from the Dyak probe set (Figures S6C–S6K). This background was considerably weaker than the signal obtained in homoplasmic Dyak tissue (Figures 5G and 5K), allowing background correction if necessary during image analysis.

We quantified heteroplasmy levels manually in 3D in single NSCs using mtDNA-smFISH and observed notable cell-to-cell variability in heteroplasmy levels, even between NSCs of the same brain (Figures 5L–5P). On average, NSC heteroplasmy levels measured by mtDNA-smFISH were slightly lower than the levels obtained by pyrosequencing of whole adult flies of the same genotype (Figures 5O–5P and S6L–S6P), consistent with previously described age-dependent increase of mutant mtDNA heteroplasmy levels.^19^ Large NSC-to-NSC heteroplasmy variability was also observed using pyrosequencing of mtDNA from FACS-sorted single NSCs with a different nuclear genomic background (Dpn::GFP, Figure S6N). Because mtDNA-smFISH allows direct *in situ* visualization of heteroplasmy, it now opens the possibility to investigate heteroplasmy shifts across different cells and tissues, eventually uncovering the underlying cellular and molecular processes controlling heteroplasmy regulation.^11^

### Purifying selection in the *Drosophila* female germline

**Figure 6:**
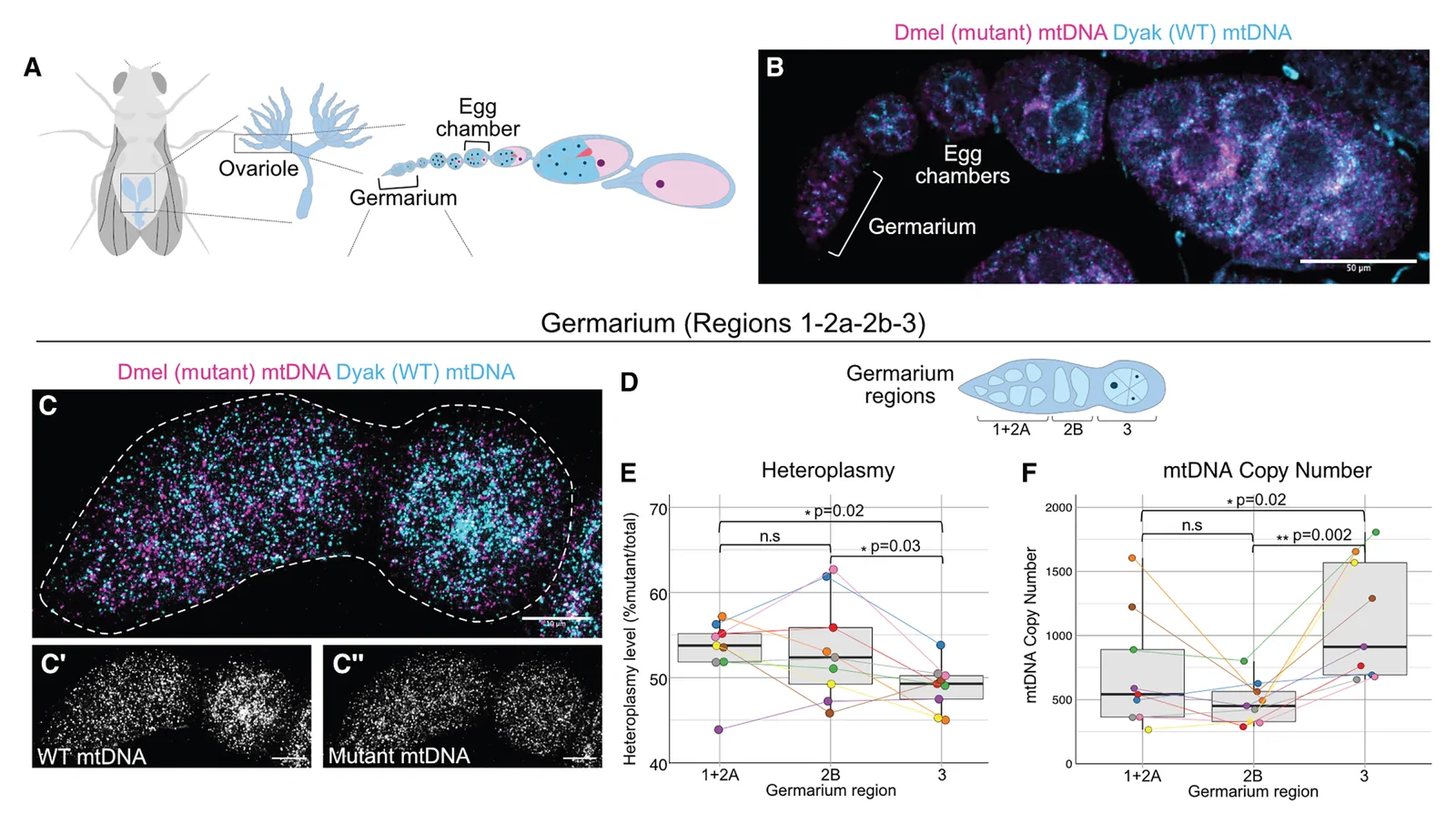
Purifying selection in the *Drosophila* female germline (A and B) Schematic of *Drosophila* oocyte development, with developing oocytes arranged in 15–20 ovarioles, linear arrays of germarium and egg chambers. (B and C–C″) Representative confocal images of mtDNA-smFISH using probes targeting only *D. melanogaster* (mutant, magenta, C″) or *D. yakuba* (WT, cyan, C′) mtDNA across the germline (B) or the germarium only (C, outline). (D) The germarium is divided into three regions that define stages of germ cell development. (E and F) Quantification of heteroplasmy level (% mutant mtDNA) (E) and total mtDNA CN (sum of Dmel and Dyak puncta) by mtDNA-smFISH across entire (3D) regions 1 + 2a, 2b, and 3, with dots indicating single Germaria (paired *t* tests or Wilcoxon signed rank tests; *n* = 9 ovaries from 7 animals). Scale bars are 50 μm (B) and 10 μm (C–C″). See also Figure S7.

Previous studies indicate that mtDNA molecules carrying deleterious mutations are subject to purifying selection during oogenesis in *Drosophila*.^39,69–72^ These conclusions were mostly based on qPCR analysis of mtDNA in homogenized ovaries and offspring from heteroplasmic female *Drosophila*,^39,69–71^ or on whole-tissue analysis with long FISH probes.^39^ However, single-cell, single-molecule analysis of this process has not been possible until recently.^41^ We used our *in vivo* mtDNA-smFISH probes to label Dmel or Dyak mtDNA in the different compartments of heteroplasmic *Drosophila* ovaries corresponding to different stages of germ cell differentiation (Figures 6A and 6B).^73^ Automated 3D quantification of mtDNA was conducted with FISH-quant.^74^ This showed a small but significant decrease in the percentage of mutant Dmel mtDNA between the regions of the germarium corresponding to stages 1 + 2a (53.1% ± 3.9%, mean ± SD) or 2b (52.0% ± 6.0%, mean ± SD) and stage 3 (48.5% ± 2.7%, mean ± SD) (*p* = 0.02 and *p* = 0.03, respectively) (Figures 6C–6E). This decrease in mutant heteroplasmy levels was accompanied by a significant increase in absolute mtDNA CN between stage 2b (477 ± 166, mean ± SD) and stage 3 (1,113 ± 468; mean ± SD; *p* = 0.002) (Figure 6F), in line with a previously proposed model whereby preferential replication of WT mtDNA allows purifying selection at this stage of germ cell development.^41,69,71,75^ When imaging entire ovaries, we also noticed instances of large cell-to-cell variability in heteroplasmy levels in the nurse cell compartments (Figures S7A–S7C). This likely indicates that the mother-to-offspring bottleneck caused by low mtDNA levels in germline stem cells (GSCs)^31^ and subsequent rapid replication during oogenesis and nurse cell formation also enhances cell-to-cell variability in heteroplasmy levels for non-oocyte GSC-derived cell types.

### A somatic bottleneck during *Drosophila* neurogenesis

**Figure 7:**
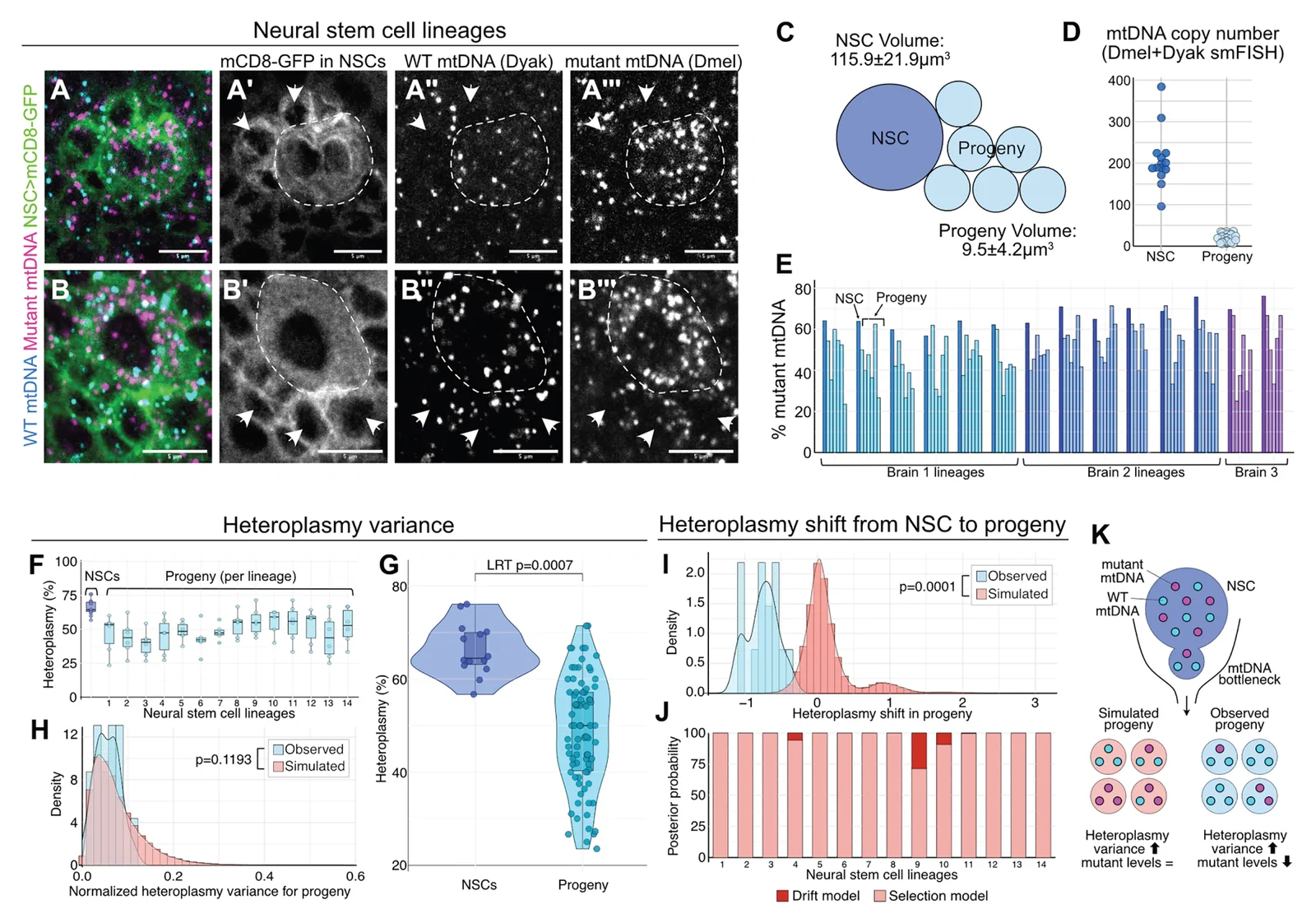
Asymmetric mtDNA segregation through a somatic bottleneck during *Drosophila* neurogenesis (A–B^″′^) Representative confocal images of NSC lineages expressing *worniu*-GAL4 > mCD8-GFP (green, A′ and B′) and mtDNA-smFISH using probes targeting only *D. yakuba* (WT, cyan, A″ and B″) or *D. melanogaster* (mutant, magenta, A^″′^ and B^″′^) mtDNA. The outline indicates the NSC, and arrows point to smaller progeny. Scale bars are 5 μm. (C) Schematic of *Drosophila* NSC lineage, with NSC volume (dark blue) on average ~12× larger than their differentiating progeny (light blue) (*n* = 6 NSCs with 4 progeny each). (D) mtDNA CN in NSCs and their progeny using mtDNA-smFISH. (E) Heteroplasmy levels quantified by mtDNA-smFISH in NSC lineages and their youngest-born progeny (identified by *worniu*-GAL4 > UAS-mCD8-GFP) (*n* = 14 NSC lineages from 3 brains from 2 experiments, as indicated). (F) Heteroplasmy levels in NSCs pooled across all lineages (dark blue) and their progeny grouped per lineage (light blue). (G) Heteroplasmy level distributions across all lineages for NSCs (dark blue) and their progeny cells (light blue). (H) Gaussian kernel density estimates of normalized heteroplasmy variance for observed progeny (light blue, *n* = 14 lineages) and progeny simulated under a random segregation model (red; 140,000 simulations). (I) Gaussian kernel density estimates of normalized heteroplasmy shift from NSCs to progeny, as observed (light blue, *n* = 14 lineages) or simulated under a random segregation model (red; 140,000 simulations). (J) Posterior probabilities for selection + bottleneck (blue) vs. pure drift (red) models across all 14 lineages. (K) Model of heteroplasmy segregation through a somatic mtDNA bottleneck during asymmetric NSC division, comparing simulated progeny (red) with observed progeny (light blue) as quantified by mtDNA-smFISH. (L) See also Figure S7.

During development, many instances occur in which large stem cells undergo asymmetric division to self-renew and give rise to a much smaller differentiating daughter cell.^76^ We asked whether this could also create a genetic bottleneck that increases variability in mtDNA heteroplasmy levels between daughter cells, similar to the pronounced heteroplasmy differences observed among offspring of the same mother.^26^ A typical example occurs during *Drosophila* neurogenesis in the developing larval brain, where we found NSCs at third instar to be on average ~12×larger than their differentiating progeny (NSCs: 115.9 ± 21.9 μm^3^; progeny: 9.5 ± 4.2 μm^3^; mean ± SD) (Figures 7A– 7C). We conducted mtDNA-smFISH with Dmel and Dyak probe sets on heteroplasmic brains expressing membrane-associated mCD8::GFP specifically in NSCs and their direct progeny (*wor-niu*-GAL4,UAS-mCD8::GFP),^60^ and performed GFP co-immuno-staining to identify individual NSC lineages (Figures 7A and 7B). Quantification of mtDNA puncta also showed an average ~12× reduction in total mtDNA molecules between NSCs and their progeny (NSCs: 208.7 ± 68.4; progeny: 16.4 ± 7.1; mean ± SD) (Figure 7D), comparable to the observed cell size reduction (Figure 7C). The average mutant heteroplasmy level was 66.4% ± 5.7% in NSCs and 48.6% ± 11.8% in progeny cells (*n* = 14 lineages from 3 brains; mean ± SD) (Figures 7E and 7F).

Using a linear mixed-effects model on logit-transformed data with NSC lineage as a random effect, we found that progeny cells showed significantly greater heteroplasmy variability than NSCs (Figures 7E–7H). A likelihood ratio test (LRT) comparing a model allowing for different variances between cell types with a null model assuming equal variances supported this increased variability (LRT = 11.39, *p* = 0.0007) (Figure 7G). We performed Monte Carlo simulation tests to evaluate whether the increased heteroplasmy variance observed in progeny could be explained by a model of random mtDNA segregation. Null distributions for normalized heteroplasmy variance were generated by randomly partitioning mtDNA molecules from a parent NSC into progeny cells. The observed increase in heteroplasmy variance among progeny cells was not significantly different from the null distribu-tion (two-tailed Monte Carlo *p* = 0.1193; Figures 7H and S7D). This supports the hypothesis that the ~12-fold reduction in mtDNA CN during asymmetric NSC division acts as a somatic bottleneck, increasing the variance in heteroplasmy levels in the progeny cells.

In addition to increased variance, the linear mixed-effects model revealed that progeny cells had significantly lower heteroplasmy than NSCs (β = −0.751, SE = 0.069, *p* < 0.001), with the odds of mutant relative to WT mtDNA in progeny cells being 0.47 times those in NSCs (Figure 7G). To test whether this could be explained by random segregation, we compared observed normalized heteroplasmy shifts with null distributions generated by Monte Carlo simulations. The observed shift was significantly different from the null distribution (two-tailed Monte Carlo *p* = 0.0001, Figures 7I and S7E), indicating that the decrease in the proportion of mutant mtDNA from NSC to progeny cannot be explained by a stochastic bottleneck alone. To quantify the strength of the putative selective mechanism, we used Approximate Bayesian Computation (Figures S7F and S7G) and found strong evidence for purifying selection, with a selection model favored over a pure drift model in all lineages (posterior probabilities of the selection model between 0.713 and 1.0; 13/14 lineages with posterior probabilities > 0.9; Figures 7J and S7H). The median selection coefficient (s) was estimated to be s = -0.51 (range -0.32 to -0.64; Figure S7H), indicating that mutant mtDNA was about half as likely as WT mtDNA to be present in progeny cells. Asymmetric NSC division and neuronal differentiation must therefore involve an as-yet-unknown mechanism of purifying selection that favors segregation or selection of functional mtDNA into neuronal progeny (Figure 7J).

## Discussion

Heteroplasmy levels for a given mtDNA mutation can show striking cell-to-cell variability, even among the same cell type within a single organ. They also change dynamically over time and thereby may contribute to aging and the pathogenesis of mito-chondrial disorders.^77^ Our understanding of the molecular mechanisms that drive or allow cell-type-specific accumulation of mtDNA mutations remains limited,^11^ largely due to the inability to efficiently visualize mtDNA sequences in specific cells within their tissue context and combine this with immunostaining and genetic manipulations. Here, we describe a robust method for visualizing mtDNA heteroplasmy in whole-mount *Drosophila* tissue and in human cells using sequence-specific smFISH of mtDNA combined with HCR for enzyme-free signal amplification. mtDNA-smFISH enables efficient detection of mtDNA in various *Drosophila* tissues, including the brain, salivary gland, wing discs, muscles, and ovaries. We show that it provides high detection efficacy (>90%) by comparing mtDNA-smFISH-based measurements of single-cell mtDNA content with single-cell ddPCR measurements of mtDNA.

Specificity was assessed in cell culture and whole-mount tissues by applying mtDNA-smFISH to comparable tissues or cells from other species (human, *D. melanogaster*, or *D. yakuba*), in Rho^0^ cells, upon RNase treatment, and upon expression of a mito-chondrial-targeted restriction enzyme to selectively degrade WT mtDNA.

Using this method, we visualized and quantified mtDNA CN and heteroplasmy levels *in vivo* in various developing *Drosophila* tissues, focusing on the female germline and the developing brain, two tissues with key importance for mitochondrial disease inheritance and presentation.^78^ In heteroplasmic *Drosophila* developing brains, we observed cell-to-cell variability in heteroplasmy levels. This was evident among individual NSCs in the same brain, in line with single-NSC heteroplasmy measurements by pyrosequencing. However, cell-to-cell variability was significantly higher among postmitotic progeny, even when derived from a single NSC. NSCs undergo asymmetric divisions^76^ to produce ~12× smaller progeny, accompanied by a proportionate reduction in mtDNA CN from ~200 to ~16 mtDNA molecules per cell. This therefore gives rise to a subcellular somatic genetic bottleneck, whereby only a subset of NSC mtDNA molecules segregates into the differentiating progeny, enhancing genetic drift and inter-neuronal heteroplasmy variability (Figure 7K). Statistical modeling of this bottleneck supports our interpretation of mtDNA CN reduction as the main factor underlying increased heteroplasmy variance in NSC progeny. This is consistent with findings in mammals demonstrating an ongoing, linear increase in mtDNA variance over time.^7,79^ Previous mechanistic studies of the germline bottleneck suggest that an increase in variance is a stochastic process caused by binomial partitioning of mtDNA molecules during cell division and random mtDNA turn-over.^80^ Many other instances of asymmetric cell division or cell size reduction occur throughout development and tissue regeneration, for example in hair follicles.^32,33^ This is likely to lead to similar increases in heteroplasmy variance and may underlie the accumulation of clonal mtDNA mutations in some neurons, as observed in the brain.^81^

The rapid segregation of mtDNA heteroplasmy, which occurs due to a genetic bottleneck, can also provide the substrate upon which purifying selection can act by exposing deleterious mtDNA mutations to subtle selection mechanisms.^27,71,82–84^ In *Drosophila*, the developing brain in larvae was previously found to show stronger purifying selection against mutant mtDNA than other tissues, such as the wing disc, gut, or the aging adult brain.^19^ We found mutant mtDNA levels to be consistently lower in progeny than in the NSC from which they directly derived, with our statistical analysis revealing signatures of purifying selection that reduce mean heteroplasmy levels in progeny cells. This suggests that neurogenesis is accompanied by an as-yet-unknown mechanism of purifying selection to ensure that newborn neurons carry more functional mtDNA, thereby limiting the potential deleterious impact of increased heteroplasmy variability caused by a somatic bottleneck. We also observed purifying selection at a well-characterized genetic mtDNA bottleneck during the early stages of *Drosophila* oogenesis. This process modulates mother-to-offspring mtDNA inheritance and has been studied extensively before, mostly through indirect measures, such as qPCR measurements of mtDNA heteroplasmy levels in offspring,^18,39,70,71,75^ and more recently through another approach for single-molecule visualization of mtDNA heteroplasmy.^41^ Purifying selection in the female germline is thought to occur through a range of mechanisms, in particular autophagy/mitophagy of mitochondria carrying pathogenic mtDNA mutations,^39,70^ and preferential protein translation on the surface of mitochondria with WT mtDNA.^71,75^ Selective mitophagy of mitochondria with damaged mtDNA has also been observed in *C. elegans*^85,86^ and human cell culture.^87^ However, within each NSC lineage, almost all progeny contained lower heteroplasmy than the NSC from which they derived. Since we quantified heteroplasmy only in the most recently born progeny, localized close to the parent NSC, it seems likely that mtDNA is already subject to asymmetric segregation of WT rather than mutant mtDNA during NSC division. This is reminiscent of previous observations in mammalian cells^88,89^ and yeast,^90–92^ showing that stem cells can selectively segregate young/old mitochondria or WT/damaged mtDNA into daughter cells. This process could be driven by the intricate molecular machinery that regulates asymmetric apico-basal apportioning of intrinsic NSC/progeny fate determinants in *Drosophila* neuroblasts.^76,93^ It will be important to confirm these observations, determine which mechanisms act in NSCs and their progeny, whether these mechanisms are conserved in other somatic tissues and among species, and whether and how recently described mtDNA quality control mechanisms, such as nucleoidophagy,^94–96^ may act specifically on mitochondria containing WT or mutated mtDNA.

Together, the insights gained from this work contribute to our understanding of mtDNA dynamics and provide a valuable tool for investigating mitochondrial inheritance and evolution in various contexts, with the potential to have broader implications for understanding human mitochondrial diseases, neurodegeneration, and aging.

### Limitations

mtDNA-smFISH cannot differentiate mitochondrial genomes that differ only by a single-nucleotide polymorphism, as several variants or small indels are needed to confer probe specificity. Precisely how much sequence divergence is required remains to be determined. Automated heteroplasmy quantification through freely accessible software (FiJi) was performed in 2D, whereas more accurate 3D quantification was done either manually or using paid software (MATLAB). Another limitation is that minor cross reactivity of *D. yakuba*-specific probes with *D. melanogaster* mtDNA was observed, which can be addressed by adjusting thresholds for signal quantification. Nonetheless, when applied in the appropriate heteroplasmic context and combined with immunostaining and targeted genetic perturbations, mtDNA-smFISH constitutes a powerful approach for probing the cellular and molecular mechanisms that shape mtDNA heteroplasmy dynamics during development and aging.

## Resource Availability

### Lead contact

Requests for further information and resources should be directed to and will be fulfilled by the lead contact, Jelle van den Ameele (jv361@cam.ac.uk).

### Materials availability

All materials, resources, and reagents are listed in the STAR Methods section and Table S1. Further requests should be directed to and will be fulfilled by the lead contact.

## Star★Methods

### Key Resources Table

**Table:** 

REAGENT or RESOURCE	SOURCE	IDENTIFIER
Antibodies		
rat anti-Deadpan	Abcam	Cat#ab195173; RRID:AB_2687586
chicken anti-GFP	Abcam	Cat#ab13970; RRID:AB_300798
mouse anti-ATP5A1	Abcam	Cat#ab14748; RRID: AB_301447
rabbit anti-FLAG M2	Cell Signalling	Cat#D6W5B; RRID:AB_2572291
guinea pig anti-heat denatured GFP	Kondo et al.^97^	N/A
rabbit anti-heat denatured GFP	Kondo et al.^97^	N/A
rabbit anti-TOMM20	Thermo Fisher Scientific	Cat#10G6I8; RRID:AB_2848869
mouse Ig2a anti-TOMM20	Santa Cruz	Cat#sc17764; RRID:AB_628381
rabbit anti-TFAM	GeneTex	Cat#GTX103231; RRID:AB_11176720
biotin-conjugated goat anti-Chicken IgY (H+L) secondary antibody	Invitrogen	Cat#PA1-28657; RRID:AB_10982216
Chemicals		
Dulbecco’s Modified Eagle Medium	Thermo Fisher Scientific	Cat#10566016
Fetal Bovine Serum	Thermo Fisher Scientific	Cat#16140071
Trypsin	Sigma-Aldrich	Cat#T2601
Prolong Diamond antifade mounting medium	Thermo Fisher Scientific	Cat#P36961
Formaldehyde	Thermo Fisher Scientific	Cat#28908
Formamide Deionized	Merck Millipore	Cat#S4117
TritonX-100	Sigma-Aldrich	Cat#STBJ7635
Tween20	Sigma-Aldrich	Cat#SLCH9711
20X SSC	Sigma-Aldrich	Cat#S6639
Hybridization solution	Molecular Instruments	Cat#BPH02023
Washing buffer	Molecular Instruments	Cat#BPW002225
Amplification buffer	Molecular Instruments	Cat#BAM02622
DNeasy Blood and Tissue kit	Qiagen	Cat#69504
QIAamp DNA Micro Kit	Qiagen	Cat#56304
Power SYBR™ Green PCR Master Mix	Applied Biosystems	Cat#4367659
Phusion HF polymerase	NEB	Cat#NEB M0530
PyroMark PCR Kit reagents	Qiagen	Cat#PN:978703
PyroMark Q48 Autoprep	Qiagen	Cat#PN:974002
Magnetic Beads PyroMark Q48	Qiagen	Cat#PN:1096484
Tris-HCl	Sigma-Aldrich	Cat#T8943
Proteinase K	NEB	Cat#P8107AA
ddPCR Supermix for Probes (no dUTP)	BIO-RAD	Cat#186-3010
Papain	Sigma-Aldrich	Cat#P4762
Collagenase	Sigma-Aldrich	Cat#C2674
Insulin	Sigma-Aldrich	Cat#10516
Penicillin and streptomycin (pen-strep)	Sigma-Aldrich	Cat#P4458
L-glutamine	Sigma-Aldrich	Cat#G7513
2’,3’-Dideoxycytidine	Sigma-Aldrich	Cat#D5782
RNase A	Roche	Cat#10109142001
Alexa Fluor 488 Streptavidin	Abcam	Cat#ab272187
Deposited data		
smFISH mtDNA copy number and cell volume data (NSC-progeny)	This paper	GitHub: https://github.com/JvdAlab/mtDNA-smFISH; Zenodo:10.5281/zenodo.18852757
Experimental models: Organisms/strains		
UAS-mito-HA-GFP	BDSC	RRID:BDSC_8442
UAS-mito-HA-GFP,e1	BDSC	RRID:BDSC_8443
UAS-Mito-XhoI-Myc	BDSC	RRID:BDSC_81007
Mi(PT-GFSTF.0)dpnMI00051-GFSTF.0	BDSC	RRID:BDSC_59755
UAS-Mito-LbNox-Flag (in attP2)	This study	N/A
*worn/u*-GAL4	BDSC	RRID:BDSC_2364
UAS-mCD8::GFP	BDSC	RRID:BDSC_5137
w^1118^;+;+ (*D. melanogaster* mtDNA)	BDSC	RRID:BDSC_5905
w^1118^;+;+ (*D. yakuba* mtDNA)	Ma et al.^68^	N/A
w^1118^; (*mt:ND2^del1^+mt:COI^T300I^;* *D. yakuba* mtDNA)	Ma and O’Farrell^18^	N/A
Oligonucleotides		
Drosophila common mtDNA-smFISH probes	This study; Molecular Instruments	Lot# PRH417; Table S1
D. yakuba mtDNA-smFISH probes	This study; Molecular Instruments	Lot# PRH253 and PRJ122; Table S1
D. melanogaster mtDNA-smFISH probes	This study; Molecular Instruments	Lot# PRH254 and PRJ123; Table S1
Human common mtDNA-smFISH probes	This study	Table S1
Human deletion mtDNA-smFISH probes	This study	Table S1
Software and algorithms		
R statistical software (v4.4.2)	R Core Team, 2024^98^	https://www.r-project.org/
Fiji (ImageJ) (v1.53f or later)	Schindelin et al.^99^	https://fiji.sc/
nlme (R package)	Pinheiro and Bates, 2000^100^	https://CRAN.R-project.org/package=nlme
EasyABC (R package)	Jabot et al.^101^	https://CRAN.R-project.org/package=EasyABC
abcrf (R package)	Pudlo et al.^102^	https://CRAN.R-project.org/package=abcrf
Analysis code and data	This paper	GitHub: https://github.com/JvdAlab/mtDNA-smFISH; Zenodo:;https://doi.org/10.5281/zenodo.18852757

### Experimental Model Details

#### Drosophila strains and husbandry

Homoplasmic *Drosophila melanogaster* strains were maintained at 25°C, and heteroplasmic strains carrying the temperature-sensitive mt.COI^T300I^ mutation at 29°C, unless indicated otherwise. The following stocks were used: UAS-mito-HA-GFP (BDSC_8442); UAS-mito-HA-GFP,e1 (BDSC_8443); UAS-Mito-XhoI-Myc (BDSC_81007); Mi(PT-GFSTF.0)dpnMI00051-GFSTF.0 (Dpn::GFP) (BDSC_59755); UAS-Mito-LbNox-Flag (unpublished). The GAL4-driver used throughout the study was *worniu*-GAL4 on II^60^ (BDSC_2364), either on its own or recombined with UAS-mCD8::GFP on II (BDSC_5137), to mark NSCs and their immediate progeny (where GFP expression perdures). Heteroplasmic *Drosophila*, carrying a combination of wild-type *D. yakuba* mtDNA and mutant *D. melanogaster* mtDNA (mt:ND2^del1^+mt:COI^T00I^), on a nuclear *D. melanogaster* w^1118^ genomic background, were described previously.^18^ Homoplasmic *Drosophila*, carrying *D. yakuba* mtDNA on a nuclear *D. melanogaster* w^1118^ genomic background, were described previously.^68^ Heteroplasmy was monitored routinely by PCR-restriction digest (see below; Figures S1I, and S1J). Apart from the experiments on ovaries, all experiments were done on both male and female larvae, but sex was not included as co-variate in the analysis.

***Drosophila melanogaster S2R+ cells*** were maintained in Schneider’s *Drosophila* Medium (Thermo Fisher Scientific 21720024) supplemented with 10% fetal bovine serum (FBS) (Thermo Fisher Scientific 16140071), in a humidified incubator at 25°C. The cell line is presumed male (FlyBase ID FBtc0000150).

***HeLa cells*** were cultured in Dulbecco’s Modified Eagle Medium (DMEM) (Thermo Fisher Scientific 10566016) with 10% FBS (Thermo Fisher Scientific 16140071) in a closed 10ml dish at 37°C, 5% CO_2_ and ambient O_2_. Medium was refreshed every other day. Upon reaching confluency (every 3-4 days), cells were detached using Trypsin (Sigma-Aldrich T2601) for 3-5 minutes, resuspended in culture medium, spun down at 200 xg for 2 minutes at room temperature (RT), resuspended in 1 ml of fresh medium, and diluted 1:10 in a fresh culture dish. The cell line is female.

***ΔH2.1 cybrid cells*** carrying a heteroplasmic mtDNA deletion (a patient-derived 7.5 kb partial deletion from m.7982 to m.15504) were obtained from Dr Carlos Moraes.^64^ The sex of the cells is not known. Cells were cultured in a 5% CO_2_ and 37°C incubator in DMEM-high glucose-pyruvate (Gibco 41966), supplemented with 10% FBS (Gibco A5256701) and 50 μg/mL uridine (Sigma U3003). The Rho^0^ cell line was derived from low heteroplasmy ΔH2.1 cybrid cells by adding 500μM of 2’,3’-Dideoxycytidine (Sigma-Aldrich D5782) to media for 17 days.

### Method Details

#### Antibodies

The following primary antibodies were used: rat anti-Deadpan (1/100, Abcam ab195173); chicken anti-GFP (1/500, Abcam ab13970); mouse anti-ATP5A1 (1/500, Abcam ab14748); rabbit anti-FLAG M2 (1/500, Cell Signalling D6W5B); guinea pig or rabbit anti-heat denatured GFP (1/500, kind gift of Hiroyuki Hioki^97^); rabbit anti-TOMM20 (1/500, Thermo Fisher Scientific 10G6I8); rabbit anti-TFAM (1/ 200, GeneTex GTX103231); mouse Ig2a anti-TOMM20 (1/200, Santa Cruz sc17764). Secondary antibodies for conventional immunostaining were Alexa Fluor 405, 488, 546, 647 -conjugated secondary antibodies raised in goat or donkey (1/500, Life Technologies). For biotin-labelled immunostaining, we used biotin-conjugated goat anti-chicken IgY (H+L) secondary antibody (1/500, Invitrogen PA1-28657) and Alexa Fluor 488 Streptavidin (1/250, Abcam ab272187).

#### mtDNA-smFISH probes

mtDNA-smFISH probes for third-generation FISH-HCR^47,49^ were either ordered from Molecular Instruments (lot numbers PRH253 and PRJ122 for Dyak-specific probes; PRH254 and PRJ123 for Dmel-specific probes; PRH417 for *Drosophila* common probes), or designed in-house (for all human probes) and ordered as desalted unmodified oligonucleotides (Sigma) (Table S1). The probe design process from Molecular Instruments is proprietary, but probes were designed to maximize sequence divergence by including at least a small indel, contained within one of the probe halves. Probes were designed against a target sequence of 52 base pairs, with two halves designed against 25 base pairs on either side, separated by two base pairs. Initiator and spacer sequences for each set of amplifiers were as in.^47,50^ Oligonucleotides were then assembled as follows: (probe half 1) Initiator1 - I1spacer - Reverse complement of 5’side of target sequence; (probe half 2) reverse complement of 3’side of target sequence - I2spacer - Initiator 2. Target sequences, and probe sequences for in-house designed probes are shown in Table S1. Probe pair number 3 was removed from the human common and deletion probe sets (Figure S5; Table S1).

#### Whole-mount mtDNA-smFISH

Larval tissues were dissected in phosphate-buffered saline (PBS), fixed in 4% formaldehyde (FA) in PBS with 0.3% TritonX-100 (Sigma-Aldrich STBJ7635) (0.3%PBSTx) for 30 minutes at RT, and washed for 3 × 5 minutes in 0.1% PBSTx at RT. Fixed tissue underwent dehydration through consecutive washes in cold PBS with 0.1% Tween20 (Sigma-Aldrich SLCH9711) (PBSTw) with 25%, 50%, 75%, and 100% methanol for 10 minutes each at 4°C. Tissue can be stored at -20°C at this stage, until *in situ* hybridization (ISH). Prior to ISH, tissues were rehydrated through consecutive washes in cold PBSTw with 100%, 75%, 50% and 25% methanol for 10 minutes each, and a final wash in PBSTw. Denaturation solution was 70% Formamide (Merck Millipore S4117), 2x saline-sodium citrate (SSC) (Sigma-Aldrich S6639) in H_2_O. Tissues were incubated in 600μl denaturation solution at 90°C in a shaking heating block at 300rpm for 10 minutes. Probes were stored at 1μM stock solution (i.e. 1μM for each probe half) at -20°C, defrosted prior to ISH, and 0.8μl was added to 200μl hybridization solution (Molecular Instruments BPH02023; stored at -20 °C), for a final hybridization concentration of 4nM for each probe-half. Final total hybridization concentration therefore depends on the number of probes in each probe set. For smaller probe sets (i.e. Dmel and Dyak specific probe sets targeting 7 and 5 sequences respectively), concentration was increased to up to 40nM, to improve signal intensity. Probes were heated in hybridization solution to 95°C for 2 minutes. Denaturation solution was removed from the tissues and replaced by the heated hybridization solution with probes. Heating block temperature was adjusted to 37°C, to rapidly but gradually cool the tissues with probes, and then incubated overnight at 37°C in a shaking heat block. In the meantime, washing buffer (Molecular Instruments BPW002225; stored at -20°C) was placed in a 37°C incubator to pre-warm. The following day (day 2), tissues were washed 4 × 15 minutes with warmed probe wash buffer at 37°C (for the first wash, wash buffer was added to the sample directly to make tissues sink to the bottom of the vial), followed by a 5 minutes wash in 50% probe wash buffer and 50% 5xSSC with 0.1% Tween20 (SSCT) at RT and two final 5 minutes washes with SSCT at RT. HCR amplifiers were defrosted in the dark, and annealed by placing 2μL (of 3μM stock for 6pmol final concentration per sample) of each hairpin in separate tubes, heated to 95°C for 1.5 minutes and cooled at RT in the dark. Next, all HCR hairpins were combined and added to amplification buffer (Molecular Instruments BAM02622; stored at 4 °C; 100μL per sample), if necessary, with nuclear counterstain DAPI (4’,6-diamidino-2-phenylindole) at 1:500, added to the tissue sample and incubated in the dark overnight at RT. The final day (day 3) consists of washes with SSCT of 2 × 5 minutes, 2 × 30 minutes, and 1 × 5 minutes, followed by 3 × 10 minutes washes with 0.1%PBSTx and 2 × 10 minutes with PBS. Tissues were mounted in ProLong Diamond antifade mounting medium (Thermo Fisher Scientific P36961). Slides were stored at 4°*C* and imaged after 24 hours.

#### mtDNA-smFISH on cultured cells

HeLa, ΔH2.1 cybrids or S2R+ cells were cultured in IBIDI 12-Well Chambers (Thistle Scientific IB-81201) in 250μL of medium, the maximum capacity for the wells. Cells were fixed using 4% FA in PBS containing 0.1% PBSTx, washed in PBSTx, and subjected to dehydration and rehydration steps as per the smFISH protocol. Only for individual probe testing (Figure S5), hybridization was performed with a final hybridization concentration of 400nM for each probe-half. For heating during the pre-hybridization step, the IBIDI plate was placed on a flat surface of an inverted heating block underneath a polystyrene cover. For probe hybridization, the IBIDI wells were placed in a wet chamber to prevent evaporation. This chamber was created by placing pre-warmed, wet tissue paper at the bottom of an empty pipette tips box, with the IBIDI wells on top. The box was maintained at 37°C in an incubator. Subsequent wash steps, and antibody staining were essentially as for the whole-mount smFISH-HCR protocol, as described above and below. RNase treatment was performed on ΔH2.1 cybrid cells after fixing and permeabilizing with PBSTx. The treatment was conducted as described previously^98^ with 1 hour incubation at 37°C in 100μg/mL RNase A (Roche 10109142001). Samples were washed three times in PBS and protocol was continued as previously described, resuming from the dehydration and rehydration steps.

#### Incorporating immunostaining with mtDNA-smFISH

For conventional antibody staining of epitopes not affected by heat denaturation (anti-Flag, anti-TOMM20, anti-ATPsyna, anti-TFAM immunostaining), or with antibodies against heat denatured GFP,^97^ immunostaining was conducted after the smFISH protocol. In the final washes, 5xSSCT was replaced by 0.3%PBSTx through serial washing with 75%, 50% and 25% 5xSSCT in PBSTx for 5 minutes each, and 3 × 5 minutes in PBSTx. For immunostaining, brains were incubated with primary antibodies in 0.1%PBSTx overnight at 4°C, washed with 0.1%PBSTx, incubated with secondary antibodies in PBSTx overnight at 4°C and washed with 0.1%PBSTx. Brains were mounted in Prolong Diamond antifade mounting medium (Thermo Fisher Scientific P36961).

For biotinylated antibody staining, primary and secondary biotinylated antibody staining followed by a second fixation step were conducted before the smFISH protocol. After the rehydration step on day 1, tissues were incubated with anti-GFP chicken primary antibody (Abcam ab13970; 1:500) in 0.1% PBSTx overnight at 4°C. The following day (day 2), tissues were washed 3 × 10 minutes with 0.1% PBSTx, and incubated overnight with goat biotinylated anti-chicken IgY (H+L) secondary antibody (Invitrogen, PA1-28657; 1:500). The next day (day 3), tissues were washed 2 × 5 minutes with 0.1% PBSTx, followed by a second fixation in 4% FA in 0.1% PBSTx for 20 minutes at RT, and washed 2 × 5 minutes in PBSTw, to next continue with the mtDNA-smFISH denaturation steps and protocol. In the final washes of the smFISH protocol, the tissues were processed as described above for conventional immunostaining, but after the final wash in 0.1%PBSTx, incubated overnight with streptavidin Alexa Fluor 488 conjugate at 1:100 (Jackson Immunoresearch 016-540-084). On the final day, 3 × 10 minutes washes were done with 0.1%PBSTx, and 2 × 10 min in PBS prior to mounting in Prolong Diamond antifade mounting medium (Thermo Fisher Scientific P36961).

#### Drosophila larval brain dissociation and fluorescence-activated cell sorting

Dissociation of *Drosophila* larval brain tissue was done essentially as described previously.^103^ About 50 L2/L3 larval brains were dissected in ice-cold PBS and transferred to an Eppendorf low binding microcentrifuge tube with 100*μ*l of ice-cold Rinaldini’s solution (800mg NaCl, 20mg KCl, 5mg NaH_2_PO_4_, 100mg NaHCO_3_, 100mg glucose in 100ml distilled H_2_O; filtered through Millipore Steriflip (pluriSelect); 10x can be stored at 4°C). Brains were centrifuged at 300 xg for 3 minutes at 4°C and the supernatant replaced with dissociation solution (445μl Schneider’s medium, 50μl papain (Sigma-Aldrich P4762) and 5μl collagenase (1mg/ml, Sigma-Aldrich C2674)). Schneider’s culture medium consists of 2.5ml FBS (Thermo Fisher Scientific 16140071), 50μl insulin (Sigma-Aldrich 10516), 500μl pen-strep (Sigma-Aldrich P4458), 2.5ml L-glutamine (Sigma-Aldrich G7513) in 18.925ml Schneider’s medium (Thermo Fisher Scientific 21720024), filtered through Millipore Steriflip (pluriSelect). Tissues were incubated at 30°C in a shaking heat block for one hour, with gentle pipetting every 15 minutes. Next, dissociated cells were centrifuged at 300 xg for 3 minutes at 4°C, washed with 1ml ice-cold Rinaldini’s solution and centrifuged at 300 xg for 3 minutes at 4°C. Subsequently, 200*μ*l Schneider’s culture medium was added, cells resuspended by 10-20 times pipetting with a 200*μ*l pipette tip, centrifuged again at 300 xg for 3 minutes at 4°C and finally resuspended in 400μl PBS with 0.04% bovine serum albmin (BSA). The cell suspension was filtered through a 10*μ*m pluriStrainer (Cambridge Bioscience 43-50010-03), after wetting the filter with 200μl PBS with 0.04%BSA.

Fluorescence-activated cell sorting (FACS) was carried out on a Melody^Tm^ Cell sorter (BD Biosciences), and data analysis was conducted on FlowJo software (BD Biosciences). The cell suspension after tissue dissociation was transferred to a 5ml 12×75mm style (Falcon) polystyrene round bottom tube with a cell strainer cap, and kept on ice before FACS. FACS settings were optimized to keep living and non-permeabilised cells (DAPI-negative and DRAGQ5) (Figures S4M and S4N) and single or specific numbers of Dpn::GFP-positive cells were collected in a 96-well PCR plate. Plates were immediately stored at -80°C for future downstream processing.

#### Droplet digital PCR (ddPCR)

ddPCR was conducted as described previously.^104^ For mtDNA CN measurements with ddPCR on sorted NSCs, cells were processed in a 96-well plate, either as single cells, or as sets of 10, 20, 50 or 100 cells. In ΔH2.1 cells, analysis was performed as single cells only. Cells were lysed in 2.5μL lysis buffer consisting of 50mM Tris-HCl (Sigma-Aldrich Trizma T8943) at pH 8.3, TWEEN-20 (Sigma-Aldrich SLCH9711), and 200μg/mL Proteinase K (New England Biolabs P8107AA). Plates were centrifuged at 1,000 xg for one minute at 4°C, incubated at 37°C for 30 minutes in a thermocycler with the lid heated at 105°C, and Proteinase K was next heat inactivated for 15 minutes at 80°C. 7.5μL of nuclease-free water was added to each sample to a final volume of 10μL, and plates were centrifuged at 1,000 xg for one minute at 4°C. The cell lysate was stored at -20°C before proceeding with ddPCR if needed. ddPCR Master mix for 1 reaction consists of 12.5μL 2x ddPCR Supermix for probes (BIO-RAD 186-3010), 1.25μL of 20x VIC or HEK (depending on the experiment), 1.25μL of 20x FAM probes and 7μL of sterile water, to a final volume of 22μL, and vortexed after preparation.

In cybrid cell lines a FAM and a HEX probe were used for MT-ND4 and MT-ND1 respectively, as previously described.^104^ PCR primer sequences (all 5’-3’) were: huND4 Forward primer AGTGCGATGAGTAGGGGAAGG; huND4 Reverse primer ACCTTG GCTATCATCACCCGAT; huND1 Forward primer GGGTTCATAGTAGAAGAGCGATGG; huND1 Reverse primer ACGCCATAAA ACTCTTCACCAAAG. Probe sequences were (all 5’-3’): huND4 (FAM) CAACCAGCCAGAACGCCTGAACGCA; huND1 (HEX) ACCCGCCACATCTACCATCACCCTC.

For *Drosophila* mtDNA CN determination with ddPCR, VIC targets both Dmel and Dyak mtDNA, while FAM targets only Dmel mtDNA (Figure S4A). PCR primer sequences were (all 5’-3’): Dyak+Dmel PCR Forward, AACAGACTTAAAATTTG AACGGCTACAC; Dyak+Dmel PCR Reverse, GGAGAGTTCATATCGATAAAAAAGATTGCG; Dmel PCR Forward, CTTCCAGTACTAGCAGGAGCTATT; Dmel PCR Reverse, CCTCCTCCCGCTGGGT. Probe sequences were (all 5’-3’): *D. yakuba* and *D. melanogaster* mtDNA (VIC), TATATCTTAATCCAACATCGAG; *D. melanogaster* mtDNA (FAM), ATCGAAATTTAAATACA TCATTTTTT. 22μL master mix was added to each well of a new 96-well plate, and 3μL of DNA from the lysed cells, or nuclease-free water to the non-template control wells to bring the total volume to 25μL. Plates were sealed, centrifuged at 1,000 xg for 1 minute at 4°C and transferred to the droplet generator. Once droplets were formed, PCR was performed for 40 cycles with denaturation at 94°C for 30 seconds and annealing and extension at 58°C for 1 minute. Finally, the 96-well plate is placed in the droplet reader for analysis. mtDNA CN was calculated as follows: mtDNA target concentration/μL x 25 × 1/fraction of total lysate input. FAM probe was specific for Dmel mtDNA (Figure S4B), and mtDNA CN calculations of Dmel mtDNA across a range of mtDNA concentrations were similar for FAM and VIC probes (Figures S4C and S4D). mtDNA CN scaled according to cell numbers (Figures S4E–S4H), further validating the assay and allowing to plot all per-cell values from these assays together. The assay variability was higher among single cells than among sets containing a group of cells (10, 20, 50 or 100), possibly due to cell doublets caused by inefficient FACS sorting, and single-cell ddPCR results were not included in the final analysis (Figures 2D, S4F, and S4H).

#### PCR restriction digest to determine Drosophila heteroplasmy

We developed a straightforward genotyping approach, to monitor Dmel and Dyak heteroplasmy levels, taking advantage of an NdeI restriction site in the Dyak mtDNA (Figures S1I and S1J), with NdeI retaining 100% in most PCR buffers. Genomic DNA was extracted from whole flies or dissected with QIAamp DNA Micro Kit (QIAGEN 56304). PCR was performed with Phusion HF polymerase (New England Biolabs, ID: NEB M0530), for 30 cycles at melting temperature of 45°C, using the following primers common to both Dmel and Dyak mtDNA: Forward 5’-TTAGACCAAATTTATTGGGAGACCC-3’ and Reverse 5’-GGAATTCCTCAACCTTTTTGTGATGC-3’, resulting in 1,283bp Dmel and 1,288bp Dyak fragments. NdeI (NEB R3131) digests the Dyak but not Dmel mtDNA, resulting in 2 smaller fragments (425 and 859bp) (Figure S1I). Heteroplasmy levels were inferred by measuring the intensity of the smaller Dyak bands vs the larger Dmel band (Figure S1J), either after imaging of the agarose gel, or on a Tapestation (Agilent).

#### Bulk and single-cell pyrosequencing of *Drosophila* mtDNA variants

In order to accurately measure heteroplasmy levels with high sensitivity in single cells, we developed a pyrosequencing approach^105,106^ to distinguish Dyak from Dmel mtDNA (Figures S6L–S6P). Pyrosequencing was done using the QIAGEN protocol, with PyroMark PCR Kit reagents (QIAGEN PN:978703), and primer design according to the PyroMark Assay Design SW 2.0 quick start guide. We determined optimal annealing temperatures for several primer pairs to allow efficient PCR amplification of the mtDNA target region. Validation was conducted through pyrosequencing of homoplasmic Dyak or Dmel animals on a standard curve of mixed PCR products (Figure S6O) and by comparing two different sets of primers (ID8 and ID9), together confirming the accuracy of heteroplasmy detection. Pyrosequencing primers (all 5’-3’) were Forward-8 TTTAATATTTGGTCCTTTCGTACT and Reverse-8 AGCCAGGTTGGTTTCTATCTTTAA, with sequencing primer GGTTTCTATCTTTAAAAAAT; Forward-9 GCTACCTTTGCACAGTCAAAATAC and Reverse-9 GTTTAAATAAAGAATTCGGCAAAA, with sequencing primer TTTGTTAAACAGGCGA. Pyrosequencing of heteroplasmic animals yielded reproducible results (Figures S6N and S6P), with values in line with our first PCR- and digestion-based method, and with an independent, previously validated qPCR-based method^67^ (Figure S6P).

Single-cell pyrosequencing was conducted on sorted NSCs (as in Figures S4M and S4N). Before processing, the 96-well plate was spun down at maximum speed (2,204 xg) at 4°*C* for 20 minutes, and samples were processed in a dedicated PCR hood with UV treated equipment. PyroMark PCR Master mix was pipetted straight onto the single-cell 96-well PCR plate, spun down for 1 minute at 300 xg and PCR was for 45 cycles with melting temperature of 62°C. 2μl of the PCR was verified on a 0.9% agarose gel, and PyroMark Q48 Autoprep (Qiagen) was used to conduct pyrosequencing of 10μl sample in a 48-well disc pyrosequencing plate with 3μl of Magnetic Beads PyroMark Q48 (PN:1096484).

#### Quantitative PCR (qPCR) of ΔH2.1 single mtDNA deletion heteroplasmy

DNA was isolated from ΔH2.1 cell lines using the DNeasy Blood and Tissue kit (Qiagen #69504) according to the manufacturer’s protocol. qPCR was performed to assess mitochondrial DNA (mtDNA) levels relative to nuclear DNA using β-actin as nuclear reference gene and ND1, COX3 and D-loop as mtDNA markers. PCR primer sequences were (all 5’-3’): β-actin Forward CACCATTGGCAATGAGCGGTTC; β-actin Reverse AGGTCTTTGCGGATGTCCACGT; ND1 Forward GGCTATATACAACTACGCAA AGGC; ND1 Reverse GGTAGATGTGGCGGGTTTTAGG; COX3 Forward AATCCAAGCCTACGTTTTCACACT; COX3 Reverse TAGGCCGGAGGTCATTAGG; D-loop Forward CACCCAAGAACAGGGTTTGT; D-loop Reverse TGGCCATGGGTATGTTGTTAA. The assay was performed using Power SYBR™ Green PCR Master Mix (Applied Biosystems #4367659) on a QuantStudio3 qPCR system. Each reaction contained 10μL of SYBR Green Master Mix, 0.5μM of each primer, and 30ng of total DNA template. The PCR thermal cycling protocol consisted of: initial denaturation at 95°C for 10 min, followed by 40 cycles of 95°C for 15 s and 60°C for 1 min. A melt curve analysis was performed at the end to confirm amplification specificity. Relative mtDNA levels were calculated using the ΔCt and normalizing to the nuclear marker. Fold change was determined as 2 ^(-ΔΔCt). All reactions were performed in technical triplicates for all three experiments.

#### Confocal and Airyscan laser scanning microscopy

Fluorescent images were acquired using a Zeiss LSM880 confocal microscope, equipped with Fast AiryScan, with a 63x oil immersion objective, as 12- or 16-bit images. ΔH2.1 and Rho^0^ cells were imaged using a 100× objective lens (NA1.4) on a Nikon Eclipse TiE inverted microscope with a 4-channel integrated laser engine (ILE-400) using an Andor Dragonfly 500 spinning disk system, equipped with a Zyla 4.2 PLUS sCMOS camera, coupled with Fusion software (Andor). All images are single sections, unless indicated otherwise. For whole-mount quantifications, slice thickness was ~0.8μm, with a 1/3 step size, ensuring complete NSCs or germaria were imaged and reconstructed. For ΔH2.1 cell quantification, seven sections of 0.2μm each were acquired. All images from the same experiment were acquired with the same parameters including exposure time and laser intensities. Images were processed for brightness and contrast using ImageJ. Images shown in the figures were smoothed once in FIJI, and intensity and contrast were set to the same levels within the same experiment.

### Quantification and Statistical Analysis

#### Image analysis

##### Cell culture mtDNA-smFISH image pre-processing

Four-channel fluorescence microscopy images were acquired containing signals for common probe-set (CP, Channel 1), deletion probe-set (DP, Channel 2), mitochondrial outer membrane marker TOMM20 (Channel 3), and TFAM (Channel 4). Image processing and quantitative analysis were performed using custom ImageJ macros. Analyses of cell-culture images were performed on maximum-intensity projections of each z-stack to consolidate near-diffraction-limited nucleoid puncta across planes, boost signal-to-noise, and avoid slice-to-slice redundancy or z-registration artefacts when quantifying spots and colocalization. Following acquisition, regions of interest (ROIs) were manually selected using polygonal selection tools to isolate individual cells or mitochondrial networks. The selected regions were cropped after clearing pixels outside the ROI boundary, ensuring analysis was restricted to biologically relevant areas while maintaining the original four-channel structure. The CP and DP channels underwent identical preprocessing to enhance nucleoid visualization. Background fluorescence was removed using a rolling ball algorithm (radius = 50 pixels), followed by initial Gaussian blur (σ = 2) to reduce noise. To sharpen nucleoid signals and improve spot detection accuracy, a custom deconvolution step was implemented using a 5×5 Laplacian kernel with centre weight of 24 and surrounding weights of -1, applied through convolution filtering with normalization. This deconvolution approach enhanced the point spread function of individual nucleoids while suppressing diffuse background fluorescence, critical for accurate quantification of discrete mtDNA foci. The deconvolved signals underwent morphological minimum filtering (radius = 2 pixels) to remove spurious peaks, followed by a final Gaussian blur (σ = 2) to smooth the enhanced features while preserving nucleoid centres. The anti-TOMM20 mitochondrial marker channel was processed with background subtraction (rolling ball radius = 50 pixels), contrast enhancement (0.35% saturation), and Gaussian blur (σ = 2) applied iteratively to generate a clean mitochondrial network signal. Binary masks were created using Otsu’s automatic thresholding to define mitochondrial boundaries. TFAM channel immunofluorescence signals underwent background subtraction with a smaller rolling ball radius (20 pixels) to preserve finer structural details, followed by iterative contrast enhancement and Gaussian blur (σ = 2). An additional round of background subtraction was applied to eliminate residual cytoplasmic signal before final contrast adjustment.

##### Cell culture mtDNA-smFISH common/deletion probes colocalization analysis within mitochondria

Processed CP and DP channels were masked with the anti-TOMM20 mitochondrial network staining to restrict analysis to mitochondrial regions. To preserve intensity information critical for accurate spot detection, two parallel versions of each masked image were maintained: intensity-preserved 32-bit images for quantitative analysis and user-adjustable thresholded versions for visualization. Spot detection utilized the Find Maxima algorithm with prominence thresholds of 15 for CP and 12 for DP, optimized for each channel’s signal characteristics. Colocalized spots were identified through coordinate-based proximity analysis, where DP spots within three pixels of any CP spot were classified as colocalized. This distance-based approach ensured robust colocalization detection without requiring pixel-perfect overlap. Normalization calculations were performed to determine the ratio of colocalized DP spots to individual channel counts. Deletion percentages representing CP nucleoids without associated DP signal were calculated as [(CP spots - colocalized dP spots)/CP spots] × 100.

##### Cell culture mtDNA-smFISH TFAM colocalization analysis

This pipeline quantified CP nucleoid association with TFAM signal. Following TOMM20 mitochondrial network masking of CP signals, an additional TFAM mask was applied using Otsu’s automatic thresholding to identify CP spots within TFAM-positive regions. Intensity-preserved 32-bit images were maintained throughout all masking operations to ensure accurate spot detection via the Find Maxima algorithm (prominence = 15). Sequential mask application first restricted CP analysis to the TOMM20-defined mitochondrial network, then further refined detection to TFAM-positive subregions within mitochondria. The percentage of CP nucleoids colocalized with TFAM was calculated as [(CP spots in TOMM20∩TFAM)/(CP spots in TOMM20)] × 100, revealing the proportion of mitochondrial CP nucleoids residing in TFAM-enriched domains.

##### Whole-mount tissue mtDNA-smFISH analysis

NSC progeny (i.e. NSC together with their latest-born progeny) were identified through perdurance of mCD8::GFP expression after activation in NSCs using *worniu*-GAL4, with cells closest to the NSC considered most-recently born.^107^ For automated quantification of heteroplasmy in NSCs in 3D, stacks were cropped to individual NSCs, and MorphoLibJ^108^ was used for segmentation of the *wor-niu*-GAL4,mCD8::GFP IHC signal, to create a mask for downstream quantification with FishQuant.^109^ Segmentation of germaria was performed manually in the X-Y axis, maintaining the entire imaging Z-stack for 3D quantification. mtDNA-smFISH puncta were quantified using FishQuant,^109^ following the standard protocol outlined in the online user manual, using either the germarium or the NSC mask as input. For cell or germarium segmentation, the “Define Outline” tool was used to manually trace individual cells using the polygon selection tool; background filtering was applied using the 3D_2XGauss method with a kernel size of 5 pixels in both the XY and Z dimensions (Kernel BGD XY,Z: 5,5), and a kernel signal-to-noise ratio (SNR) of 0.5 in both XY and Z (Kernel SNR XY,Z: 0.5,0.5). Spot detection thresholds were then manually optimized to ensure accurate puncta selection, with lower thresholds capturing more background signals. Once an appropriate threshold was established, the selection and parameters were saved for consistent application across samples, to maintain comparability between datasets, and applied as batch mode for other replicates. When NSCs were difficult to segment, we instead used the CellCounter plugin in FiJi^99^ to count mtDNA puncta manually. For *worniu*-GAL4 x UAS-mitoGFP or UAS-mitoXhoI experiments, NSCs were delineated based on GFP-expression or nuclear morphology (DAPI) and mtDNA spots quantified manually; NSCs not expressing *worniu*-GAL4^107^ were determined based on typical location within the VNC. Occasionally, Gaussian blur was applied to NSC images prior to segmentation or quantification.

##### Signal and background quantification

Similar areas of the larval ventral nerve cord were selected in ImageJ/FiJi,^99,110^ or entire ΔH2.1 and Rho^0^ cell images were saved as.tiff files, and imported into Matlab (The MathWorks Inc.), where the number of pixels at a given intensity value in 256 bins was quantified. Intensity distribution graphs are shown at a log scale to enhance differences in the lower values.

##### Data output and visualization

All pipelines generated binary masks, spot detection overlays, and quantitative results including total spot counts, colocalization percentages, and normalized ratios. Processed images and numerical results were automatically saved, with spot detection visualizations provided for quality control and validation of the automated counting algorithms. Graphs were made using R Statistical Software (v4.1.2; R Core Team 2021),^111^ and figures were compiled in Affinity Designer.

#### Statistical analysis and modelling

For each experiment, the number of brains analyzed ranged from 1-10. Each experiment where statistical analysis was performed included at least three biological replicates in cell culture experiments, and at least two in *Drosophila* experiments. Prism (GraphPad Software) and Excel (Microsoft) were used to perform statistical tests for Figures 2H; 6E, 6F; S2G: Shapiro-Wilk and F-tests to verify normality and variance, and t-tests, Wilcoxon signed rank, or Mann Whitney U test with Holm-Bonferroni multiple comparisons correction to determine significance.

Differences in heteroplasmy variance between NSCs and progeny were statistically tested using a linear mixed-effects model to account for non-independent measurements from the same cell lineage. Heteroplasmy percentages were first converted to proportions (*h*) and then logit transformed using the formula *logit*(*h*) = ln (*h*/(1 – *h*)) after squeezing values of 0 and 1 to ε and (1-ε) respectively, where ε = 1/(2N_max) and N_max is the maximum observed mtDNA CN, to satisfy the assumptions of normality and constant variance. The models included cell type (NSC or progeny) as fixed effect and cell lineage as random effect. A likelihood ratio test (LRT) was performed to compare a null model assuming equal variances (Model 0) to an alternative model allowing for different variances for each cell type (Model 1), implemented with the ‘varIdent’ structure in the ‘nlme’ R package.^100^

$$\;\text{Model}\;0.\;(\text{equal}\;\text{variances}\;\text{for}\;\text{NSC}\;\text{and}\;\text{progeny}):\;logit\;\left(h_{ij}\right)=\beta_{0}+\beta_{1}\cdot {\left(\;cell\;type\;\right)}_{jk}+u_{i}+\varepsilon_{ij},\;\text{where}\;u_{i}\sim N\left(0,\sigma_{u}^{2}\right)\;\text{and}\;\varepsilon_{ij}\sim N\left(0,\sigma^{2}\right)$$

$$\;\text{Model}\;1.\;(\text{unequal}\;\text{variances}\;\text{for}\;\text{NSC}\;\text{and}\;\text{progeny}):\;logit\;\left(h_{ij}\right)=\beta_{0}+\beta_{1}\cdot {\left(cell\;type\right)}_{jk}+u_{i}+\varepsilon_{ij},\;\text{where}\;u_{i}\sim N\left(0,\sigma_{u}^{2}\right)\;\text{and}\;\varepsilon_{ij}\sim N\left(0,\sigma_{k}^{2}\right)$$

Here, *i* indexes cell lineage, *j* indexes individual observations, *k* indexes cell type of observation *j. u*_*i*_ represents the random intercept for lineage *i* and *ε*_*ij*_ represents the residual error with variance $\sigma_{k}^{2}$ specific to cell type *k* in Model 1. For the LRT, both models were fitted using Maximum Likelihood (ML). Parameter estimates (*β*-coefficients and standard errors) were extracted from the best-fitting model, which was subsequently refitted using Restricted Maximum Likelihood (REML) for unbiased estimation. Statistical significance was assessed at the *α* = 0:05 level. We used Monte Carlo simulations to test whether observed heteroplasmy dynamics were consistent with a null model of random mtDNA segregation. For each of the NSC lineages, we simulated partitioning of mtDNA from the parent NSC into its set of progeny cells 10,000 times, using hypergeometric sampling, which models sampling without replacement and thus accounts for the progressive reduction of the parental mtDNA pool. The size of each draw was set to the experimentally observed mtDNA copy number of the corresponding progeny cell. The normalized heteroplasmy variance and shift were calculated for both observed and simulated data. We calculated normalized heteroplasmy variance for a cell-population with heteroplasmies *h* = _(_ *h*_2_, …, *h*_*n*_) where *n* is the number of cells in the population and mean heteroplasmy $\bar{h}$ as $V^{\prime }(h)=Var(h)/(\bar{h}(1-\bar{h}))$.^112^ The normalized heteroplasmy shift from an NSC to a progeny (Δ*h*′) was calculated as $\text{Δ}=ln\left(\frac{h_{Progeny\;}\left(1-h_{NSC}\right)}{\left(1-h_{Progeny\;}\right)h_{NSC}}\right)$.^113^

To generate test statistics comparable to the observed data, we calculated the mean normalized heteroplasmy variance and shifts across all NSC lineages for each simulation iteration. This produced null distributions of 10,000 values for each test statistic under the assumption of random segregation. Statistical significance was assessed by calculating two-tailed p-values as *p* = (*k* + 1) / (*n* + 1), where *k* is the number of simulated statistic values that deviated from the null distribution mean by atleast as much as the observed statistic value, and *n* = 10,000 is the total number of simulations. The standard errors on the null distribution means were 1.32× 10^−4^ for normalized heteroplasmy variance and 9:02 × 10^-4^ for normalized heteroplasmy shift, confirming adequate precision for reliable inference.

We used Approximate Bayesian Computation (ABC) to perform formal model comparison between a pure drift and a selection model and to quantify the strength of selection required to explain the reduction in heteroplasmy levels observed in progeny cells. Two competing models were simulated, a pure drift model (s = 0) based on hypergeometric sampling, identical to the null model used in the Monte Carlo tests; and a selection model where the parental mtDNA pool was weighted by fitness based on a selection coefficient (s ≠ 0), prior to sampling. Summary statistics for the ABC analysis were calculated on both observed and simulated data and a set of five summary statistics (mean heteroplasmy, mean normalized heteroplasmy shift, median heteroplasmy and 25^th^ and 75^th^ percentiles of heteroplasmy level) were selected for their high information content using a random forest-based approach with the ‘abcrf’ R package.^102^ The same uniform prior U(-1, 0.1) was used for both model selection and subsequent parameter estimation. Model choice was performed by training a random forest classifier (abcrf::abcrf) using a reference table generated from 10,000 simulations (5,000 per model), with a forest of 500 trees. The ‘predict’ function was then used to calculate the posterior probability of each model for the observed data based on the proportion of votes from the trees in the forest. For parameter estimation, the posterior distribution of the selection coefficient (s) was estimated for each lineage individually using the “Lenormand” ABC-SMC algorithm^114^ implemented in the ‘EasyABC::ABC_sequential’ R package.^101^ The analysis was run with 1,500 particles and an adaptive tolerance scheme set by a minimum acceptance rate of 0.02 and alpha = 0.5. Uniform prior of *U*[−1, 0:1] for the selection coefficient was used. To validate the model’s goodness-of-fit, we performed a posterior predictive check. For each lineage, we simulated a full dataset using parameters drawn from its posterior distribution and calculated the 95% credible intervals for each of the five summary statistics. The observed summary statistics fell within their corresponding 95% credible intervals 94.3% of the time, demonstrating a well-calibrated model. Posterior median estimates and 95% credible intervals were calculated from the accepted particles, and lineages were classified as showing significant selection if the credible interval did not contain zero.

## Supplementary Material

Supplemental information can be found online at https://doi.org/10.1016/j.devcel.2026.03.011.

Supplemental information

Supplemental Table S1

## Data Availability

Image analysis and statistical modeling code and data are publicly available on GitHub (https://github.com/JvdAlab/mtDNA-smFISH) and deposited on Zenodo (10.5281/zenodo.18852757).

## Highlights

- mtDNA-smFISH detects mitochondrial DNA in cell culture and intact tissues
- Sensitive and specific single-molecule visualization of mtDNA heteroplasmy
- Neurogenesis creates a somatic bottleneck, increasing genetic variance in neurons
- Purifying selection during neurogenesis and oogenesis limits harmful mtDNA variants

## In brief

Chandrasegaram et al. directly visualize mitochondrial DNA inside intact cells and tissues and reveal how genetic cell-to-cell variability arises during development. They show that somatic bottlenecks during differentiation can increase the mitochondrial mutation load in some cells, whereas purifying selection helps to limit this during *Drosophila* brain and germline development.
