# Supplementary material for: Single-molecule mitochondrial DNA imaging reveals heteroplasmy dynamics shaped by developmental bottlenecks and selection in vivo
Source: Dev Cell. Author manuscript; Available in PMC 2026 Oct 16. (PMC7619573; doi:10.1016/j.devcel.2026.03.011)
Supplement: Supplementary file 1 [file EMS219612-supplement-Supplemental_information.pdf]

Developmental Cell, Volume *61*

Supplemental information

Single-molecule mitochondrial DNA imaging reveals heteroplasmy dynamics shaped by developmental bottlenecks and selection *in vivo*

Rajini Chandrasegaram, Sara Gottardo, Abhilesh Dhawanjewar, Antony M. Hynes-Allen, Beitong Gao, Suvagata Roy Chowdhury, Luis-Carlos Tábara, Michele Frison, Stavroula Petridi, Patrick F. Chinnery, Julien Prudent, Hansong Ma, and Jelle van den Ameele

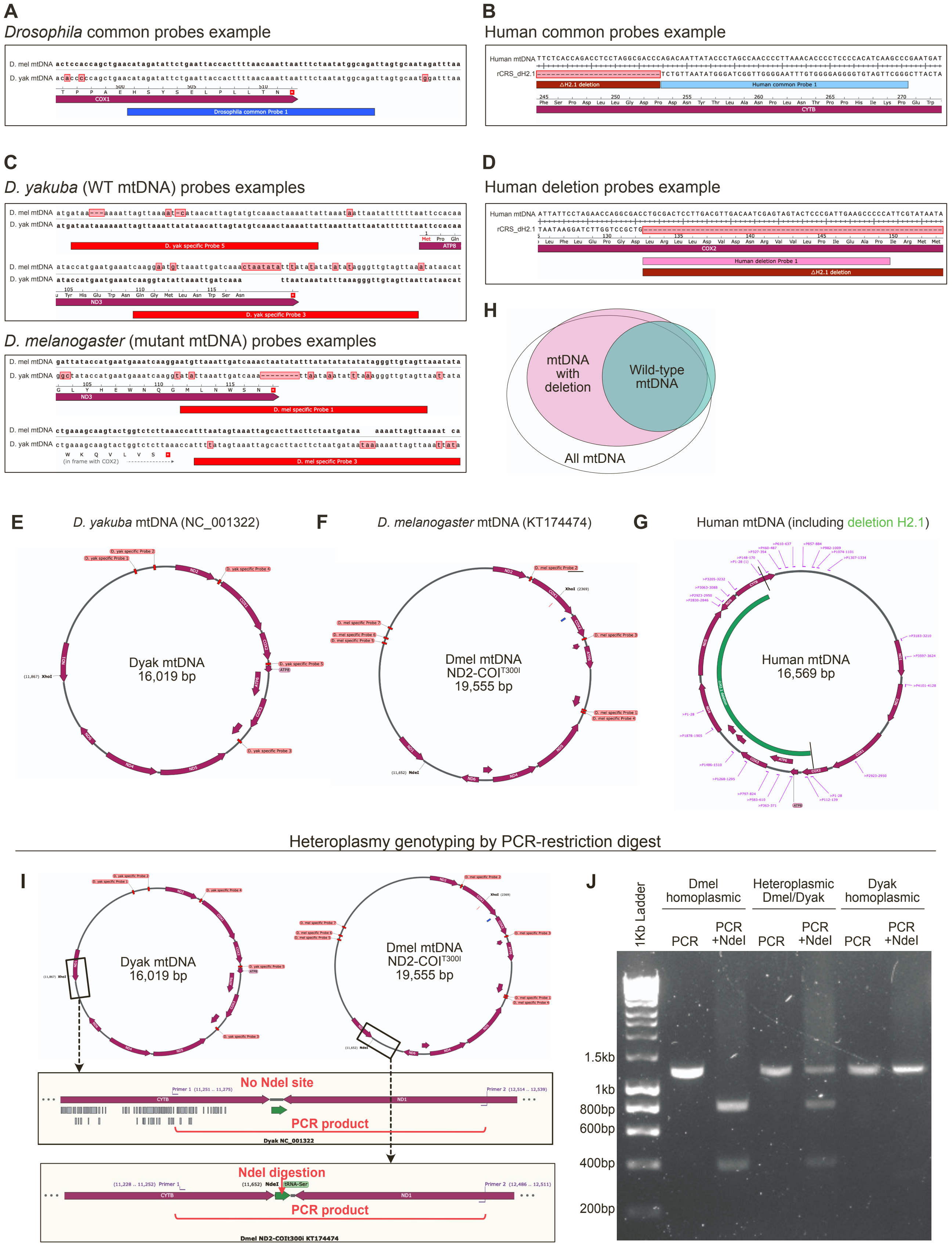

Supplementary Figure 1: Probe Design for mtDNA-smFISH of *Drosophila* and Human mtDNA, Related to Figure 1. (A-D) Representative examples of target sequences designed for *Drosophila* mtDNA, common to both *D. melanogaster* and *D. yakuba* (A), specifically for *D. yakuba* (WT, C) or

D. melanogaster (mutant, C) mtDNA, or for human mtDNA common to WT and deleted mtDNA (B) or only recognizing the WT mtDNA (D). (**E-G**) Schematic of *D. yakuba* (E), and *D. melanogaster* (F) mtDNA and human mtDNA (G) with protein-coding genes in purple and probe target sequences in bright red or pink. Human deleted sequence is indicated in green (G). (**H**) Schematic of how mtDNA-smFISH can distinguish between WT or deleted mtDNA, with deleted molecules only targeted by common mtDNA probes (red) and WT molecules also targeted by deletion-specific probes (green). (**I,J**) Schematic (A) and representative gel image (B) of PCR amplification and NdeI digestion to distinguish *D. yakuba* (WT) from *D. melanogaster* (mutant) mtDNA, with *D. yakuba* lacking the NdeI site. PCR amplification produced a 1,284bp (*D. melanogaster*) or 1,289bp (*D. yakuba*) product, which, upon digestion with NdeI, resulted in two bands of 425bp and 859bp for the *D. melanogaster* genome only.

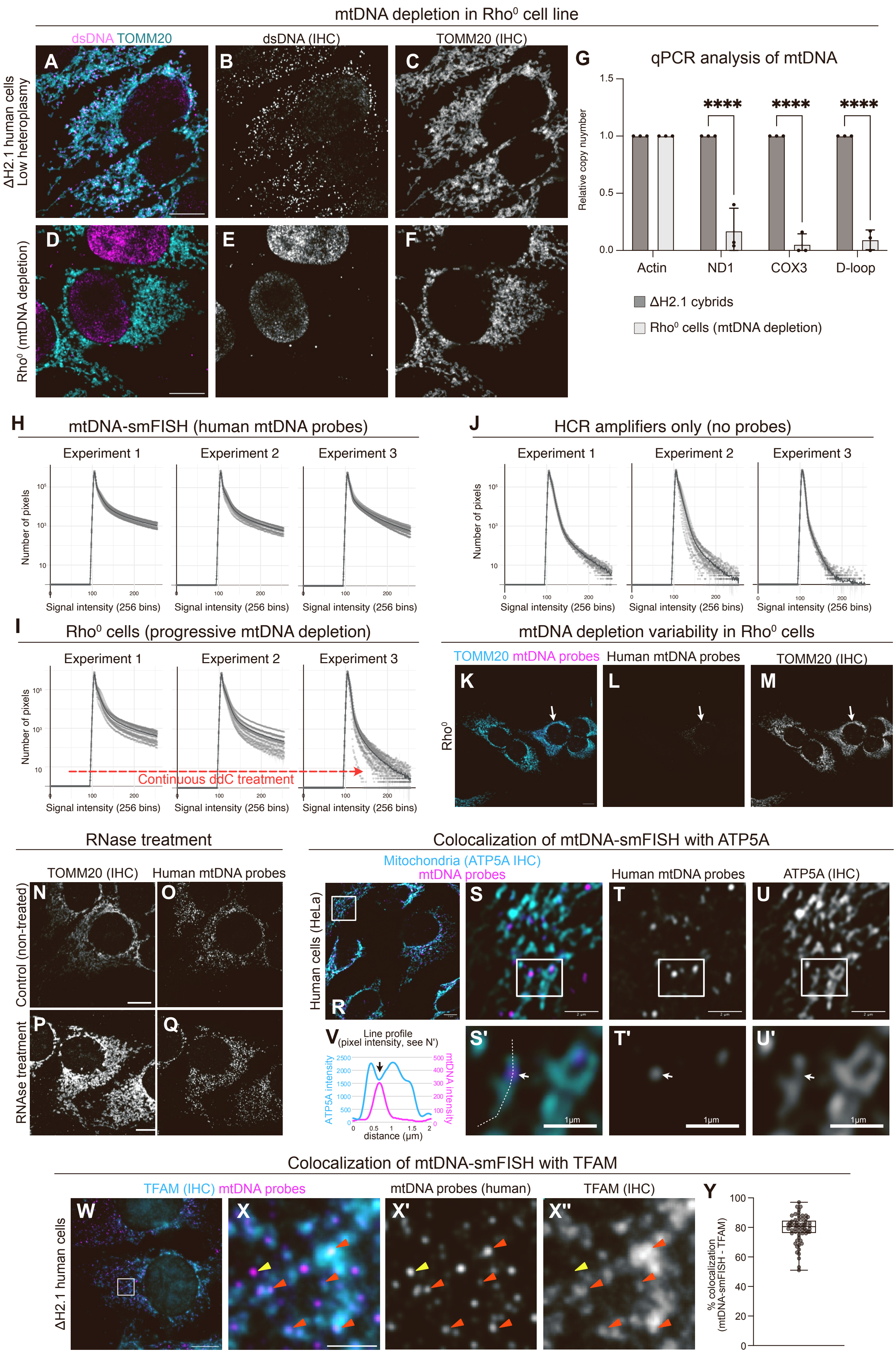
mtDNA depletion in Rho⁰ cell line
dsDNA TOMM20
dsDNA (IHC)
TOMM20 (IHC)
ΔH2.1 human cells
Low heteroplasmy
Rho⁰ (mtDNA depletion)
G
qPCR analysis of mtDNA
Relative copy nuymber
Actin
ND1
COX3
D-loop
ΔH2.1 cybrids
Rho⁰ cells (mtDNA depletion)
H
mtDNA-smFISH (human mtDNA probes)
J
HCR amplifiers only (no probes)
Experiment 1
Experiment 2
Experiment 3
Number of pixels
Signal intensity (256 bins)
I
Rho⁰ cells (progressive mtDNA depletion)
Continuous ddC treatment
mtDNA depletion variability in Rho⁰ cells
TOMM20 mtDNA probes
Human mtDNA probes
TOMM20 (IHC)
Rho⁰
RNase treatment
TOMM20 (IHC)
Human mtDNA probes
Control (non-treated)
RNAse treatment
Colocalization of mtDNA-smFISH with ATP5A
Mitochondria (ATP5A IHC)
mtDNA probes
Human mtDNA probes
ATP5A (IHC)
Human cells (HeLa)
V
Line profile
(pixel intensity, see N')
ATP5A intensity
mtDNA intensity
distance (μm)
1μm
Colocalization of mtDNA-smFISH with TFAM
TFAM (IHC) mtDNA probes
mtDNA probes (human)
TFAM (IHC)
ΔH2.1 human cells
Y
% colocalization
(mtDNA-smFISH - TFAM)

Supplementary Figure 2: mtDNA-smFISH with human mtDNA probes, Related to Figure 1. (**A-F**) Representative confocal images of immunostaining (IHC) against dsDNA (magenta, B,E) and TOMM20 (cyan, C,F) in a low heteroplasmy ΔH2.1 cybrid cell line (A-C) and a corresponding mtDNA depleted Rho^0 cell line (D-F) generated by ddC treatment. (G) qPCR analysis using ND1, COX3 and D-loop as markers for mtDNA (n = three independent experiments; unpaired t-tests). (**H-J**) mtDNA-smFISH signal (H,I) or background (J, no probes, only HCR amplifiers) quantification in ΔH2.1 cybrid cells upon continuous treatment with control (H) or ddC (I) to generate Rho^0 cells. Experiment 3 is shown in Figure 1Q. (**K-M**) mtDNA-smFISH and TOMM20 co-immunostaining in Rho^0 cells, where some treated cells retain some mtDNA (arrowhead). (**N-Q**) mtDNA-smFISH and TOMM20 co-immunostaining without (N,O) or with (P,Q) prior RNase treatment. (**R-U**) Airyscan imaging of HeLa cells after mtDNA-smFISH with human mtDNA probes (magenta) with co-immunostaining of ATP5A (cyan), with higher magnification of boxed regions (S-U) in (S'-U'). (**V**) Line profile analysis of signal along the hatched line in U'. (**W,X**) mtDNA-smFISH with human mtDNA probes (magenta) with co-immunostaining against the nucleoid marker TFAM (cyan), with higher magnification of boxed region (X,X',X''). Arrowheads illustrate mtDNA-smFISH puncta that colocalize (red) or not (yellow) with TFAM signal. (**Y**) Colocalization quantification of human mtDNA probe signal with TFAM staining after masking with TOMM20; n = 60 cells analysed across three independent experiments. Scale bars are 10μm (A,D,K,N,P,W), 2μm (S-U,X), 1μm (S'-U').

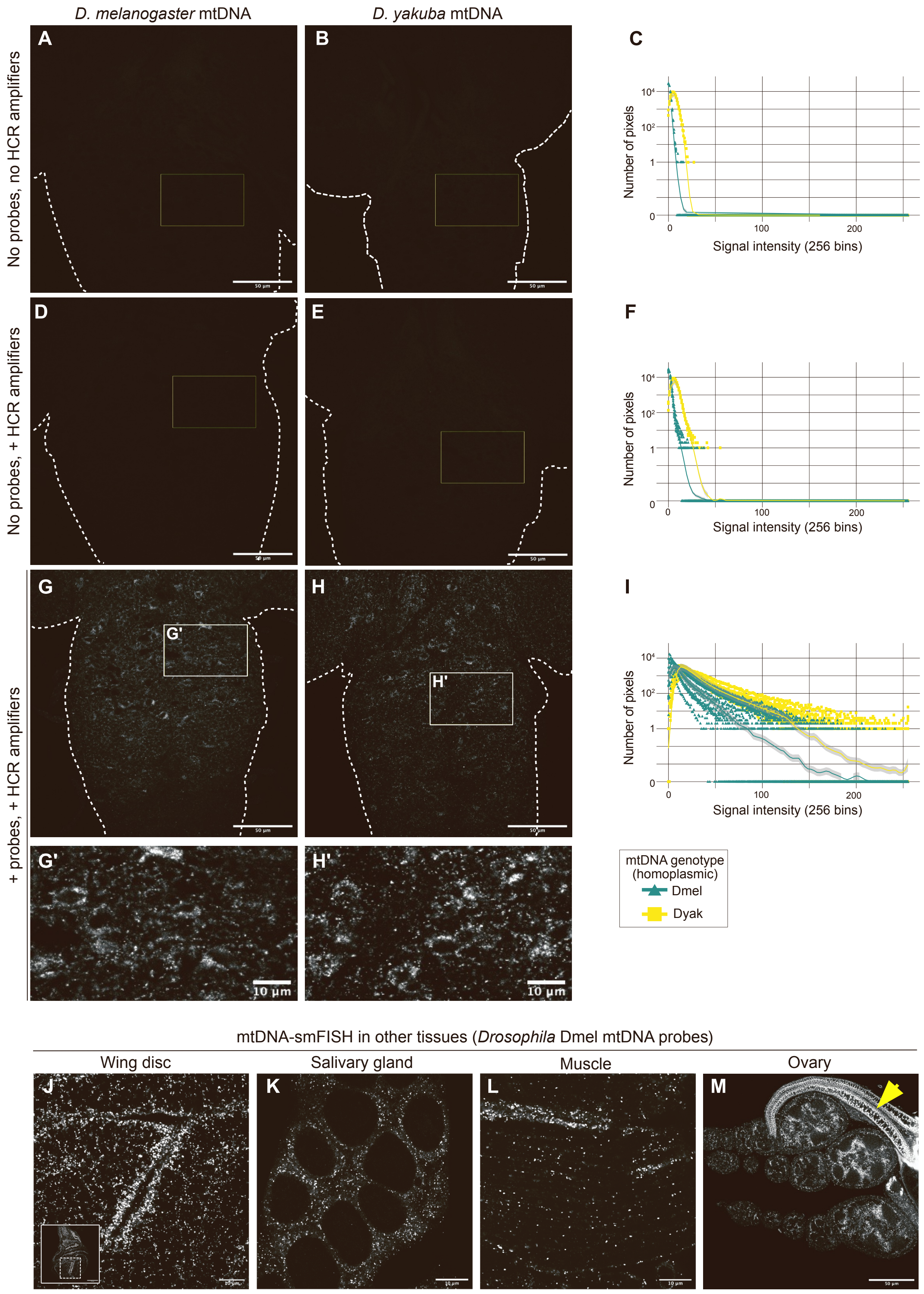

Supplementary Figure 3: mtDNA-smFISH with probes common to *D. melanogaster* and *D. yakuba* mtDNA, Related to Figure 2. (A-I) Representative confocal mtDNA-smFISH images (A-H) and signal quantification (C,F,I) with probes common to both *D. melanogaster* and *D. yakuba*

mtDNA in brains of larvae homoplasmic for *D. melanogaster* (A,D,G) or *D. yakuba* (B,E,H) mtDNA, either without probes or amplifiers (A,B,C), without probes (D,E,F), or with both probes and amplifiers added (G,H,I). (G’,H’) are high magnifications of areas boxed in G,H. (**J-M**) mtDNA-smFISH across various *Drosophila* tissues. Red arrow in (M) points to the dorsal appendage of the maturing egg. Scale bars are 50µm (A,B,D,E,G,H,J-M) or 10µm (G’,H’).

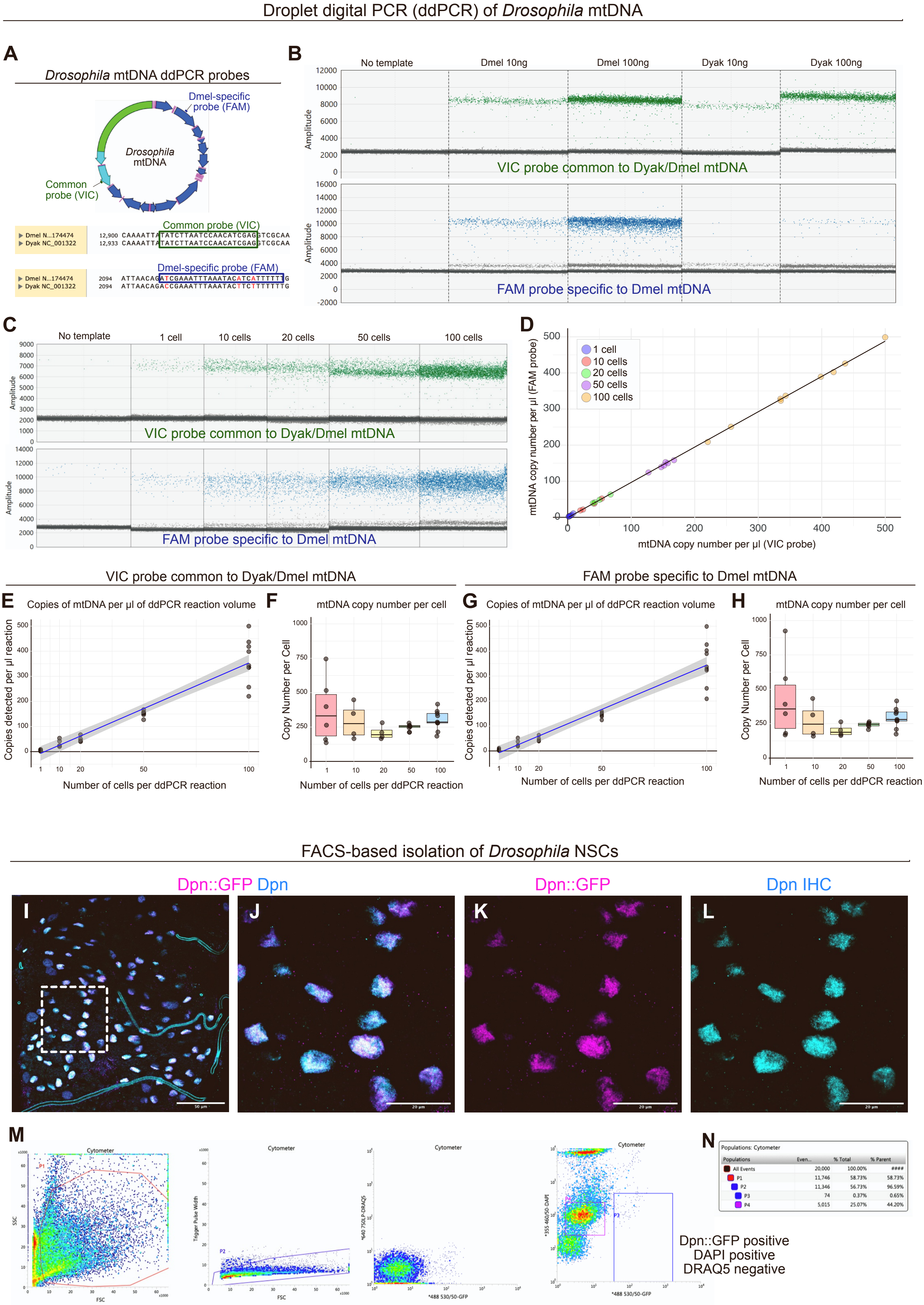

Supplementary Figure 4: Droplet digital PCR (ddPCR) for *Drosophila* mtDNA and FACS-Based Isolation of NSCs, Related to Figure 2. (A) Schematic representation of VIC and FAM

probes for *Drosophila* mtDNA. VIC probe was designed to target both *D. melanogaster* and *D. yakuba* mtDNA. FAM probe was specific to *D. melanogaster* mtDNA. (**B,C**) ddPCR using VIC (green) and FAM (blue) probes using 0, 10 or 100ng of genomic DNA from homoplasmic *D. melanogaster* or *D. yakuba* (B), or on 1, 10, 20, 50 or 100 sorted NSCs (Dpn::GFP) from homoplasmic *D. melanogaster* (C). (**D**) Correlation between mtDNA CN estimates of VIC and FAM probes on sorted NSCs from homoplasmic *D. melanogaster*. (**E-H**) mtDNA CN measured using VIC probe (E,F) or FAM probe (G,H) on 1, 10, 20, 50 or 100 sorted NSCs from homoplasmic *D. melanogaster*. mtDNA CN increases linearly with increasing cell number (E,G), but larger variability is observed in single-cell samples (F,H). (**I-L**) Immunostaining of Dpn (cyan) and GFP (magenta) of third instar larval brain from transgenic *Drosophila* with GFP fused to the NSC-marker Dpn (Dpn::GFP). (**M,N**) Representative FACS plots of dissociated third instar larval brains from Dpn::GFP *Drosophila*, with Draq5 and DAPI used to assess viability, resulting in ~0.37% of all cells retained as NSCs (N). Scale bars are 50μm (I) or 20μm (J-L).

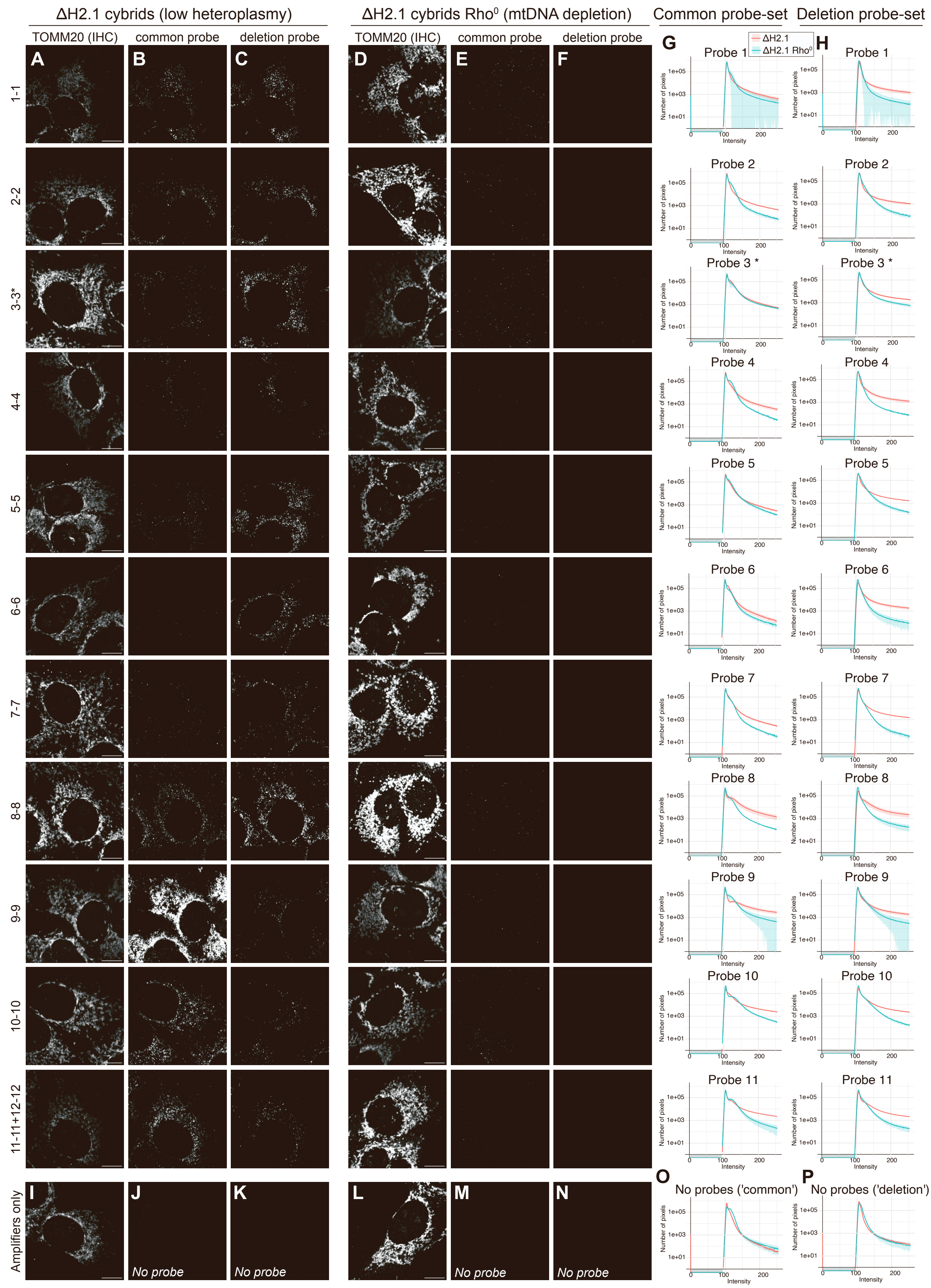

Supplementary figure 5: Specificity of individual human probes for mtDNA-smFISH, Related to Figure 4. (**A-E**) Representative confocal images of mtDNA-smFISH performed on a low

heteroplasmy ΔH2.1 cybrid cell line (A-C, I-K) and a Rho^0 (D-F, L-N) cell line by using single probes for human mtDNA (common, B,E) or human mtDNA with a single deletion (C,F). Co-immunostaining was performed with anti-TOMM20 antibody (A,D,I,L). Each row represents a single probe from each probe set. The last row (I-N) displays very low background given by the amplifiers when no probes are added during hybridization step. Signal quantification is shown in graphs (G,H,O,P). Probe 3 from the common probe set indicated with an * displayed a high background and was removed for further experiments; probe 3 from the deletion probe set was arbitrarily removed from this set as well to keep equal probe numbers. Scale bars are 10μm.

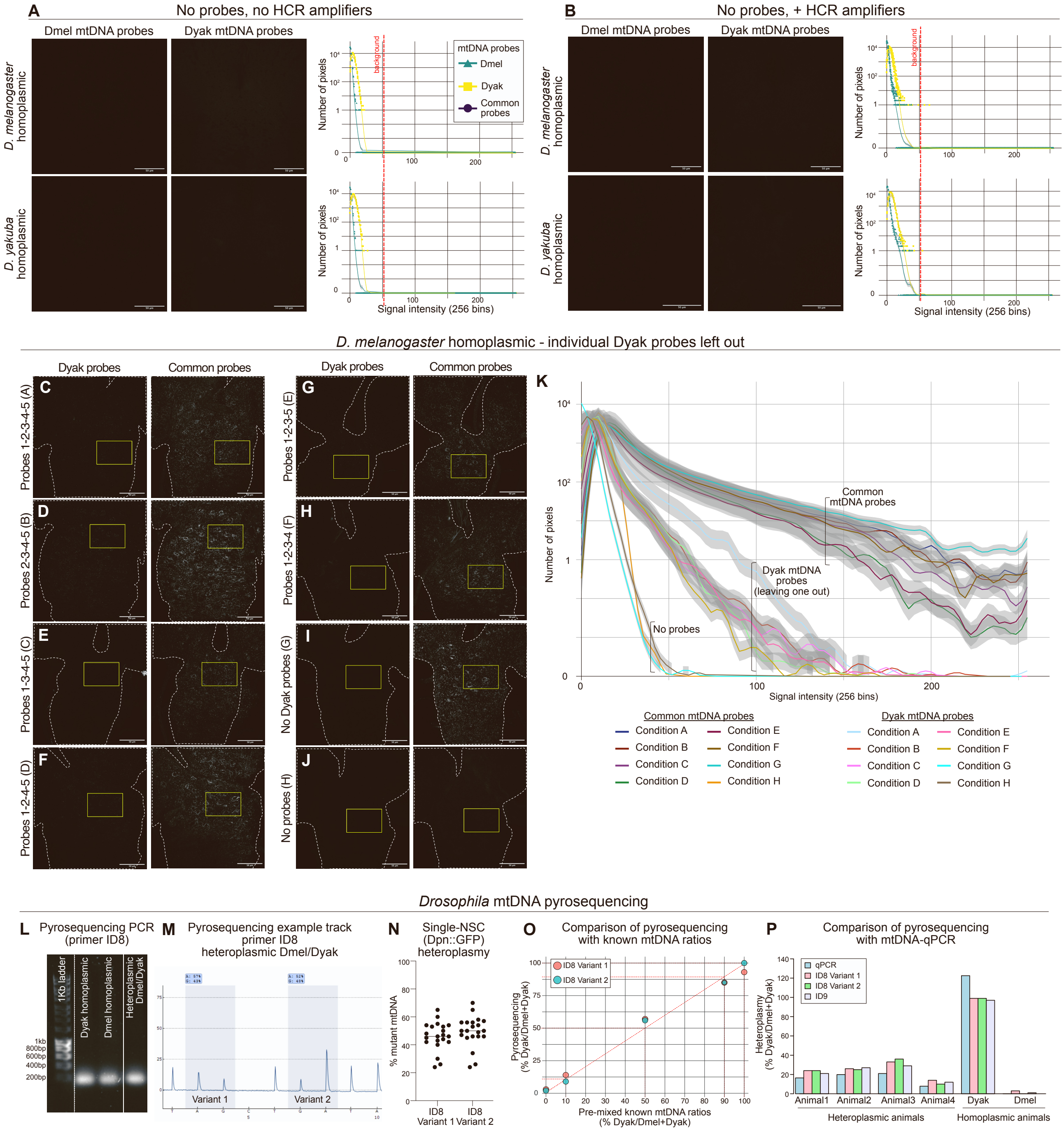

Supplementary figure 6: Specificity of *D. melanogaster* or *D. yakuba* mtDNA-smFISH, Related to Figure 5. (**A,B**) mtDNA-smFISH images and signal quantification (mean ± s.e.m.) with probes specific to either *D. melanogaster* (mutant, green graphs) or *D. yakuba* (yellow graphs) mtDNA in brains of *Drosophila* larvae homoplasmic for *D. melanogaster* or *D. yakuba* mtDNA, either without probes or amplifiers (A), or without probes with amplifiers only (B). The red dashed line in the graphs separates true signal from background, with a threshold set at 55 bins. The condition with both probes and amplifiers is shown in Figure 5D-K. (**C-K**) To assess background of the probe-set specific to *D. yakuba* mtDNA, each of the five individual probe pairs was removed separately from the probe mix used for mtDNA-smFISH on brains with homoplasmic *D. melanogaster* mtDNA. Removing

individual *D. yakuba* probes (representative confocal images in C-J, light colours in K), all resulted in comparable background levels. mtDNA-smFISH with the common probe set was used as a positive control (representative confocal images in C-J, dark colours in K). No background or signal was observed in absence of *D. yakuba* probes (I) or any probe (J). (**L-P**) Optimization of pyrosequencing to distinguish *D. yakuba* (WT) from *D. melanogaster* (mutant) mtDNA, using primer pairs ID8 (L-P) or ID9 (P). PCR using primer pair ID8 amplifies mtDNA from homoplasmic or heteroplasmic *Drosophila* strains with comparable efficiency (L), and allows quantification of heteroplasmy levels using two independent variants in the same PCR fragment (M). Pyrosequencing detects heteroplasmy levels in single NSCs sorted from Dpn::GFP third instar larvae (N). Pyrosequencing detects known ratios of mixed mtDNA from homoplasmic *D. melanogaster* and *D. yakuba* (O), and detects heteroplasmy levels from heteroplasmic or homoplasmic *Drosophila* with comparable accuracy to a previously validated qPCR method [67] (P). Scale bars are 50µm.

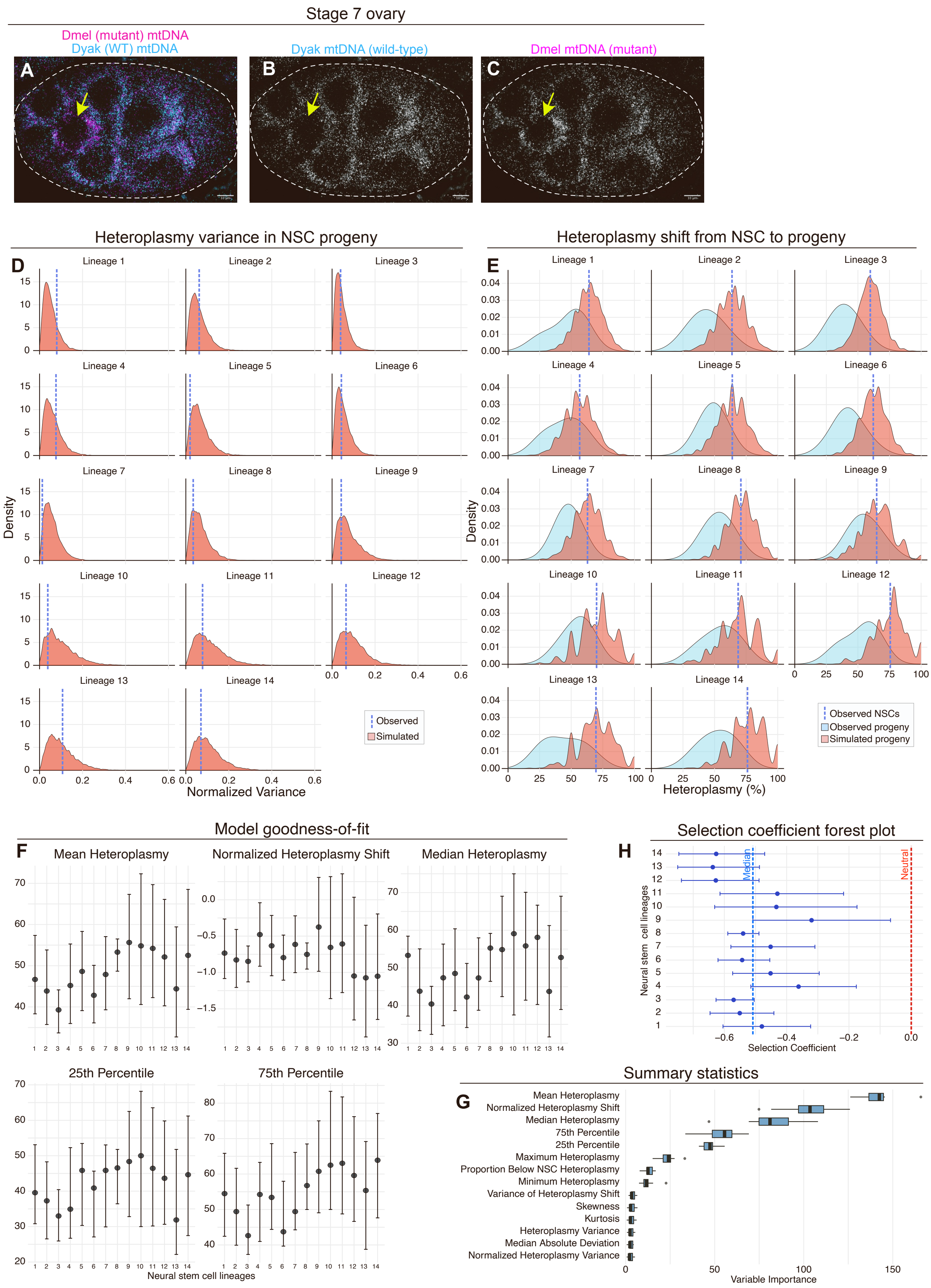

Supplementary Figure 7: Modelling of mtDNA segregation through a somatic bottleneck, Related to Figure 6,7. (**A-C**) Representative confocal images of mtDNA-smFISH using probes targeting only *D. melanogaster* (mutant, magenta, C) or *D.yakuba* (WT, cyan, B) mtDNA in stage 7

Drosophila egg chamber nurse cells. Arrow indicates a nurse cell with high mutant heteroplasmy level. Scale bars are 10μm. (**D**) Null distributions for normalized heteroplasmy variance from 10,000 random segregation simulations (red density plots) for each lineage. The corresponding observed normalized variance is marked by the dashed blue line. (**E**) Density plots of the observed progeny heteroplasmy (light blue) overlaid on the null distributions from 10,000 random segregation simulations (red) for each lineage. The heteroplasmy of the parent NSC for each lineage is marked by the dashed dark blue line. (**F**) Model goodness-of-fit assessed via posterior predictive check, comparing observed summary statistics (black dots) against 95% credible intervals of posterior predictive distribution (whiskers). (**G**) Random Forest Variable importance of candidate summary statistics for ABC parameter estimation across all 14 lineages, ranked by median importance. (**H**) Selection coefficient estimates for all lineages from Sequential Monte Carlo ABC; Points show posterior median estimates with 95% credible intervals (whiskers); blue dashed line median selection; red dashed line neutral evolution.
